# Development of a His-Tag-mediated pull-down and quantification assay for G-quadruplex containing DNA sequences†

**DOI:** 10.1039/d4cb00185k

**Published:** 2024-11-28

**Authors:** Enrico Cadoni, Hanne Moerman, Annemieke Madder

**Affiliations:** a Organic and Biomimetic Chemistry Research Group, Department of Organic and Macromolecular Chemistry, Ghent University Krijgslaan 281-S4 9000 Gent Belgium Annemieke.madder@ugent.be Enrico.cadoni@ugent.be

## Abstract

In this study, we developed a simple pull-down assay using peptide nucleic acids (PNAs) equipped with a His-Tag and a G-quadruplex (G4) ligand for the selective recognition and quantification of G4-forming DNA sequences. Efficient and specific target recovery was achieved using optimized buffer conditions and magnetic Ni–NTA beads, while quantification was realized by employing the enzyme-like properties of the G4/hemin complex. The assay was validated through HPLC analysis and adapted for a 96-well plate format. The results show that higher recovery can be achieved using His-Tag with Ni–NTA magnetic beads as compared to the more common biotin–streptavidin purification. The inclusion of the G4-ligand as an additional selectivity handle was shown to be beneficial for both recovery and selectivity.

## Abstract 2

We developed a pull-down assay using His-Tag-containing PNAs equipped with a G-quadruplex (G4) ligand for selective recognition and quantification of a DNA target in a 96-well plate format, exploiting the DNAzyme properties of the G4–hemin complex.

## Graphical abstract

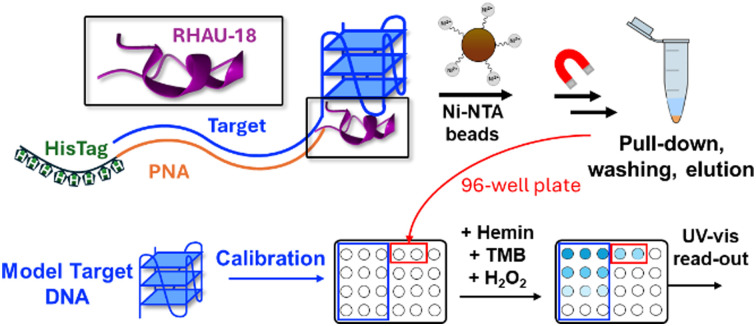

## Introduction

The sequence-specific base-pairing of natural oligonucleotides has been extensively explored for the selective recognition and detection of biomarkers, leading to the development of numerous biosensors.^1,2^ Examples of such applications include the detection of miRNAs, lncRNAs, bacterial and viral nucleic acid material, and a series of important biomarkers.^3–6^ For this purpose, probes consisting of canonical DNA can be used, even if degradation in biological media due to enzyme susceptibility (to exonucleases and endonucleases), sometimes imposes limitations.^7^ Furthermore, several nucleic acid analogues have been developed that allow formation of more stable duplexes.^8^ Among the various oligonucleotide analogs that can be used as capture probes for this purpose, peptide nucleic acids (PNAs) stand out due to their intrinsic enzymatic resistance, ease of synthesis using well-established solid-phase peptide synthesis (SPPS) procedures (which enable easy and automated decoration of PNA probes directly on solid support), and the exceptional stability of their duplexes with target sequences.^9,10^

One interesting property of DNA and RNA oligonucleotides is their ability, under certain conditions, to fold into non-canonical structures such as G-quadruplexes (G4s).^11^ These secondary structures, typical for guanine-rich sequences, are formed by the assembly of four guanines into a G-tetrad and the subsequent stacking of multiple tetrads into the final G4.^12^ This non-canonical nucleic acid structure can arise either from a single (unimolecular G4s) or multiple oligonucleotide sequences (multimolecular G4s) and it can adopt different structural conformations, including parallel, antiparallel, and various mixed topologies, often showing topological polymorphism. Due to their increasing therapeutic importance, G4 structures have been targeted by thousands of small molecule ligands,^13–15^ as well as DNA and oligonucleotide derivatives allowing sequence selective recognition.^16–18^ Moreover, G4s have been used both as a target for pull-down applications^19,20^ and as a template to direct the synthesis and mediate the pull-down of new G4-ligands.^21–23^ Besides their putative roles in many cellular processes, ranging from gene expression, telomere maintenance, RNA maturation, and aging,^11,12,24–26^ G4s can, under certain circumstances, behave as DNAzymes in the presence of hemin, an iron-containing porphyrin found in the hemoglobin, exhibiting a peroxidase-like function.^27^ This effect seems to be more efficient for parallel-shaped quadruplex structures, and it requires the docking of the hemin group on top of the G4-structure external tetrad, and the presence of H_2_O_2_ for catalyzing the oxidation of substrates, such as 3,3′,5,5′-tetramethylbenzidine (TMB). This interesting property has been extensively exploited for detection purposes, acting as a substitute for horseradish peroxidase and enabling enzyme-free colorimetric detection.^28^ In previous related work G4-forming sequences have been used as sensing probes, and similarly, G4 oligonucleotides have been added to the pulled-down material, enabling colorimetric detection.^29,30^

Many recent reports highlight the prevalence of guanine-rich tracts in relevant nucleic acid sequences that are considered biomarkers for pathologies and are therefore interesting to isolate and quantify, both in coding and non-coding RNAs.^11,31^ Concerning the latter, examples include lncRNA,^32,33^ miRNA (including their precursors pre- and pri-miRNA),^34,35^ piRNA.^36^ These oligonucleotide sequences of interest naturally exhibit a handle able to fold (under suitable conditions) into a G4 structure. In this work, we aimed to exploit that property and develop a simple pull-down assay based on the sequence-specific recognition of a DNA or RNA sequence of interest that presents a G-rich overhang allowing for quantification through the formation of a DNAzyme upon complexation with hemin. This could allow for simultaneous sequence-specific pull-down as well as quantification of the target sequence thanks to the peroxidase-like properties of the resulting complex.

**Fig. 1:**
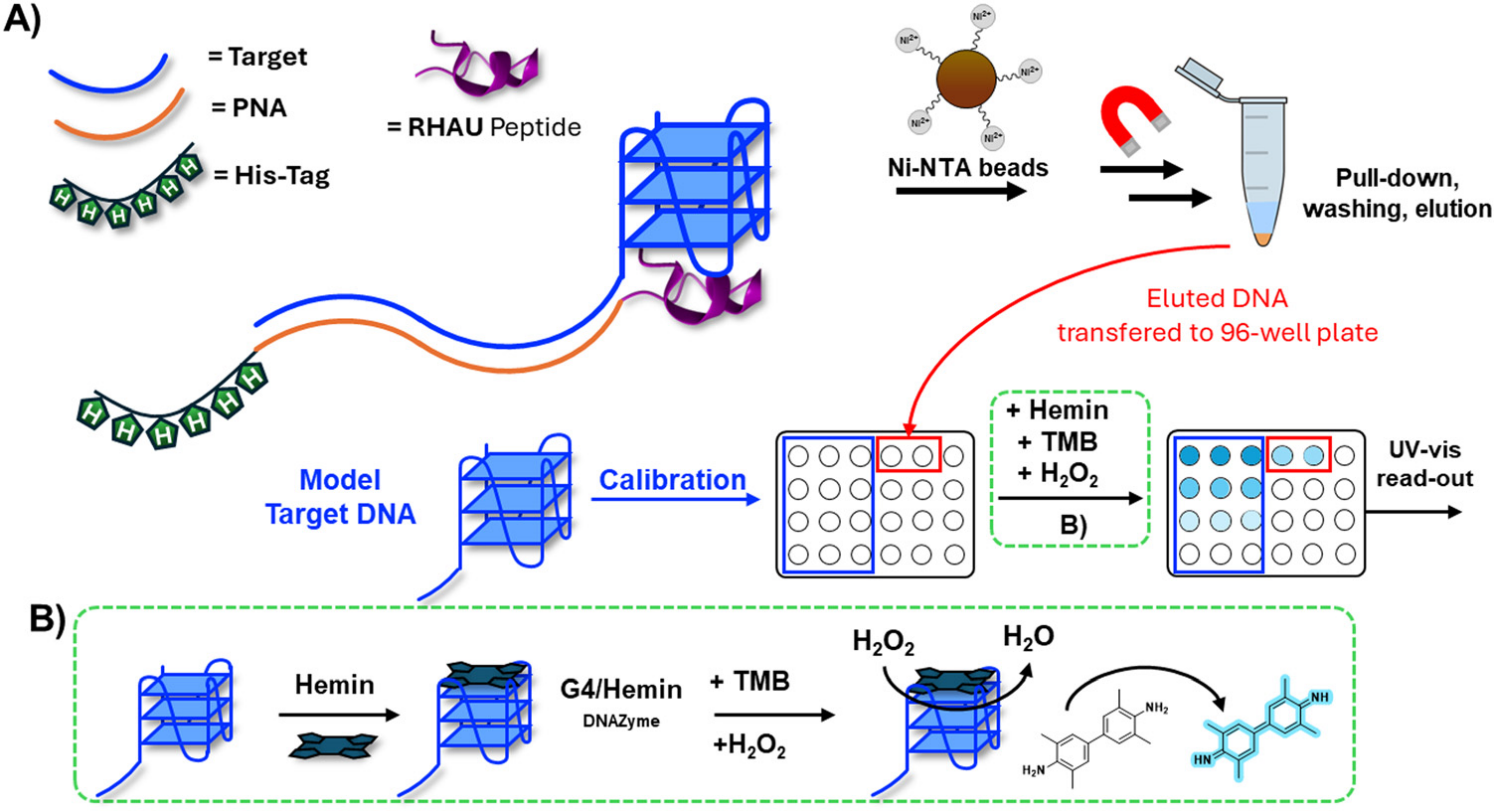
Work-flow of the assay for the pull-down and target-mediated colorimetric detection of G4-forming DNA sequences. (B) Representation of the assay work-flow and depiction of the probes used in this work. (B) Mechanism of the G4-DNAzyme peroxidase in presence of hemin, applied to the oxidation of TMB.

To achieve this, a PNA sequence was equipped with a tag (in the current work a biotin or histidine-tag) to allow for the pull-down of the target sequence upon recognition, and with a G4-ligand to stabilize the secondary structure and discriminate between a G4-forming sequence tag and a mutated, non-G4-forming control, thus increasing the selectivity of pull-down. After initial quantification of the isolated material through chromatographic techniques (HPLC-UV), we developed a simple assay based on a 96-well plate format that enables target identification and hemin/G4-based quantification using only a simple UV-plate reader (Fig. 1). In this work, for a matter of stability of the nucleic acid during sample handling, we mainly focused on DNA samples, but we foresee to expand the methodology to relevant RNA sequences in the near future.

## Results

### Choice of the purification tag

In a first, preliminary set-up to select the most suitable pull-down tag, some of the commonly used tags for protein and nucleic acid purification were exploited. Amongst the available tools for the isolation of DNA and proteins, ligand-coated iron oxide magnetic beads were selected, which allow for magnetic decantation after capture of the sample of interest. Next to streptavidin beads (SAv) also nickel II–nitrilotriacetic acid (Ni–NTA) beads which are both commercially available were tested, enabling the selective binding of biotin- or hexahistidine-tagged species respectively.

**Fig. 2:**
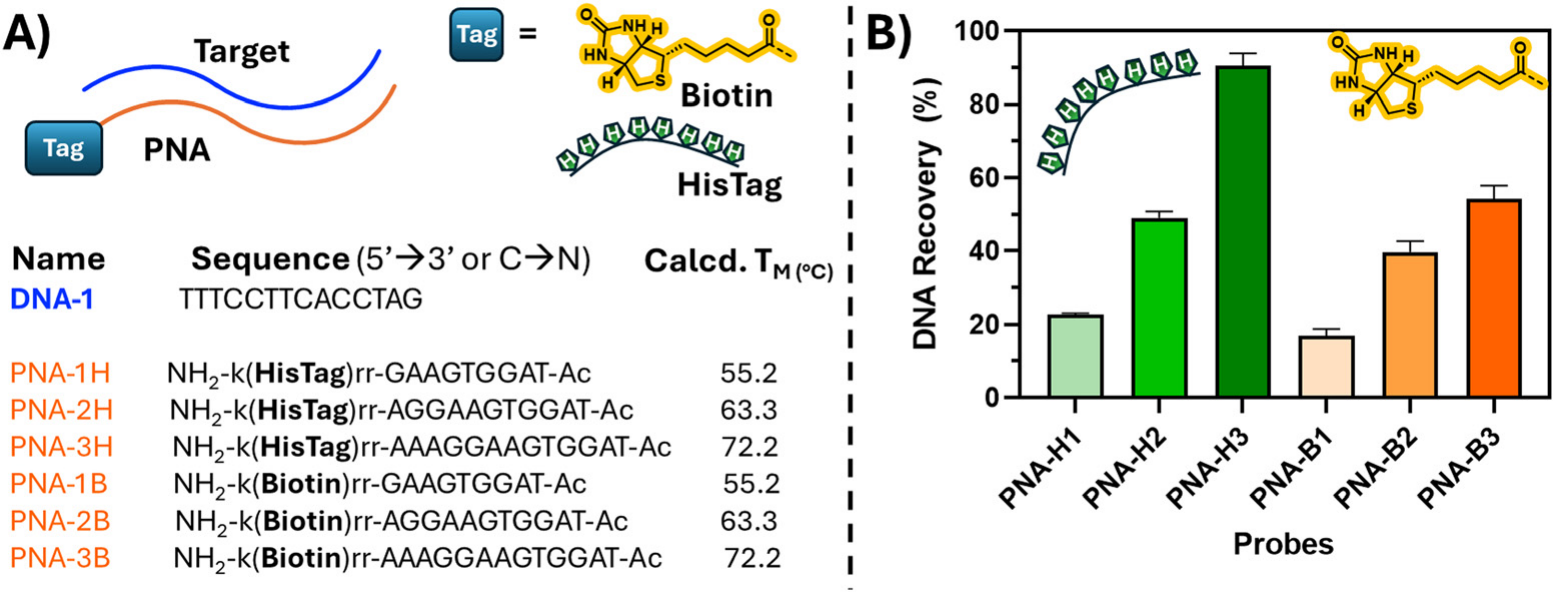
Structures and sequences of the PNA probes used in the preliminary optimization of the assay (A) and pull-down results obtained comparing the DNA recovery with biotinylated probes *vs.* His-Tag containing probes (B). The experiments were performed at 5 μM strand concentration in Tris–HCl buffer 10 mM (0.5 M NaCl, pH 7.8).

First, the recovery of a random linear DNA sequence was studied using a simple, complementary PNA probe, equipped with a biotin tag or a hexahistidine tag at the C-terminal position. In this preliminary experiment, the recovery of the target DNA was directly compared for the two tags using SAv or Ni–NTA beads respectively, excluding other factors that might interfere with the process and complicate the analysis (including the formation of secondary DNA structures and their binding with ligands included on the probe). The pull-down experiment was performed according to the provider instructions. Notably, the elution was performed differently for the two tags. For the biotin-containing probes, the samples were heated up to 90 °C to ensure the denaturation of the SAv (and the release of the material from the beads). For the HisTag probes, the samples were eluted using an imidazole-containing elution buffer, able to displace the histidine tag from the immobilized Ni–NTA ligand on the beads. Using a 5 μM strand concentration for PNA probe and DNA target sequences in Tris–HCl buffer (10 mM, 100 mM NaCl, pH 7.8) and SAv/Ni–NTA beads, we quantified the recovery *via* HPLC analysis by integrating the corresponding signals related to the recovered DNA. We tested PNA strands of different lengths (9, 11 and 13 mer for each tag), complementary to a chosen ssDNA sequence (Fig. 2(A)). Notably, DNA recovery increased progressively with increasing length of the PNA probes (from 9 to 13 mer), which can be linked to enhanced duplex stability and reduced material loss during washing. Furthermore, a generally higher DNA recovery was noticed for Ni–NTA bead-based pulldown of His-Tagged material (Fig. 2(B), green bars *vs.* orange bars). Nearly quantitative DNA recovery was observed with a 13mer PNA equipped with a His-Tag. Given the better pull-down results as well as the lower cost of Ni–NTA beads, the His-Tag-mediated pull-down was preferred over the biotin-mediated pull-down with SAv beads and ultimately selected for further optimization.

### Synthesis and design of the probes

**Fig. 3:**
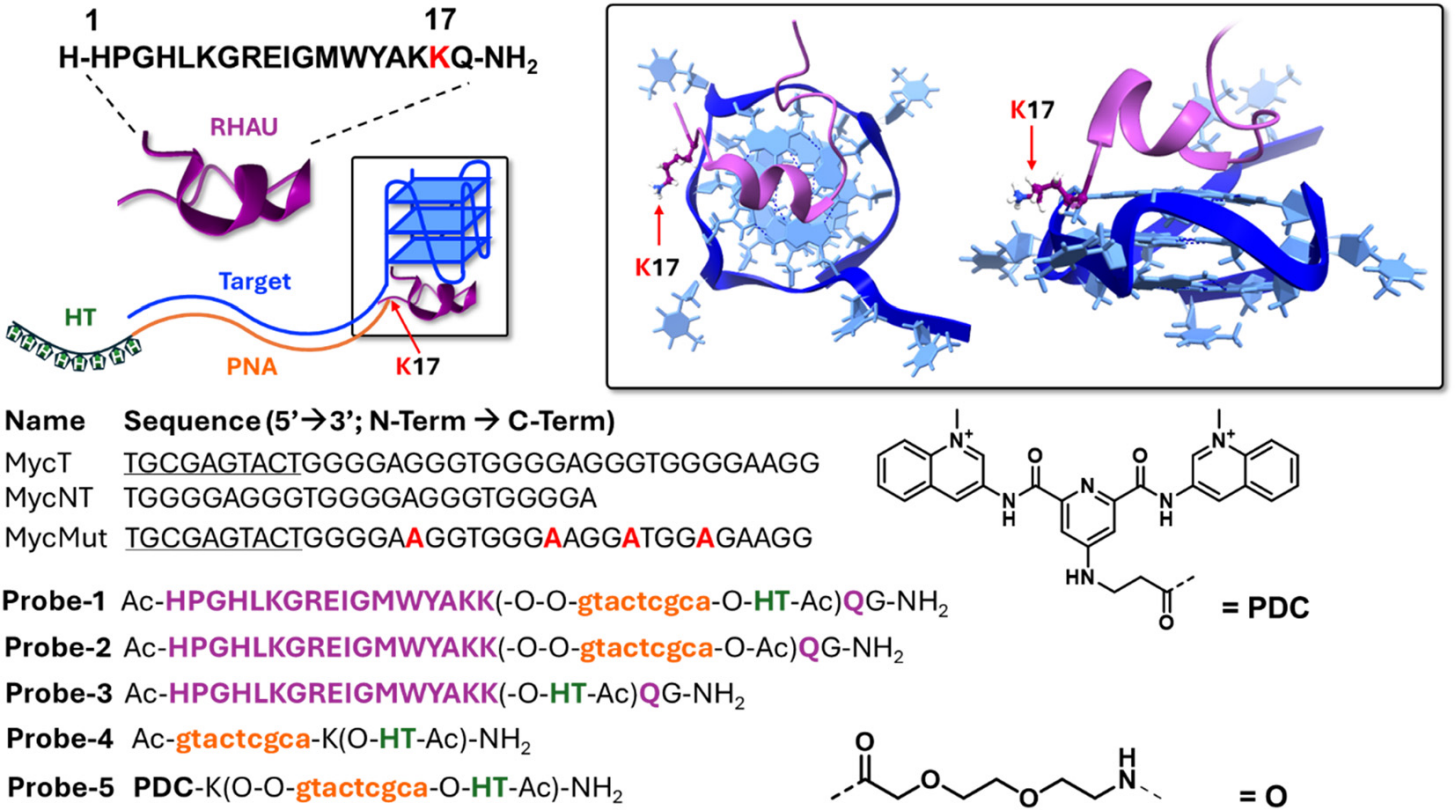
Design and sequence of the ligand containing probes and targeted DNA sequences used in this optimization process. The Rhau structure was extrapolated from the NMR solution structure reported by Phan *et al.*^38^ adapted from the PDB structure PDBID 2N21.

As a next step, more complex PNA probes, bearing the His-Tag and additionally equipped with a G4-ligand to target a G4-forming sequence downstream of the targeted DNA region, were designed. Given the compatibility between peptide and PNA synthesis through SPPS, the PNA of interest was equipped with the RHAU18 peptide (sequence HPGHLKGREIGMWYAKK), reported to selectively bind parallel G4-structures.^37–39^ Based on the reported solution structure of the peptide in complex with a parallel G4 structure (PDB ID: 2N21 and 2N16) and the known literature on the amino acid residues required for binding, we attached the PNA sequence to the side chain of lysine K17. This residue is not essential for the peptide binding to a G4 structure, and points outwards from the quadruplex core, making it ideal for the purpose reported here (Fig. 3). The core of the peptide was synthesized first, followed by growing the PNA chain on the lateral side chain of K17. For the purpose, Dde was chosen as orthogonal protecting group for this lysine residue. After Dde deprotection, the PNA was grown, and modified at the terminal position with a His-Tag. In the following experiments, the PNA length was kept short (9-mer) to prevent quantitative pull-down by the PNA alone, enabling us to evaluate the contribution of the G4 binder. Mini-PEG linkers separated each modular element (PNA, ligand, and His's-Tag, Probe-1).

As controls, we synthesized Probe-2 (lacking a tag for pull-down to verify non-specific Ni–NTA bead binding), Probe-3 (RHAU-peptide as G4-ligand, with a C-terminal His-Tag but without PNA to monitor aspecific G4 pull-down mediated by ligand-DNA interactions), Probe-4 (PNA sequence with His-Tag to verify pull-down in the absence of G4-binding element), and Probe 5 (analogue of Probe-1, with the G4-binding element replaced by the small-molecule ligand PDC/360A, synthesized according to previous reports to include a terminal carboxylic acid group for the conjugation with the PNA probe^40,41^). The molecule is reported to be a strong G4-stabiliser, and has been already used in a variety of applications related to the G4 targeting.^42–44^

All the probes used in this study were synthesised using machine-assisted SPPS procedures. The target sequence was arbitrarily chosen and used as the flanking region of the G4 sequence of cMYC DNA (MycT). Additional DNA controls included MycNT, containing the same G4-forming core but a different flanking region, and MycMut, which features the same flanking region as MycT but with a mutated non-G4-forming core.

### Pull-down optimization

#### Optimization of buffer and salt

**Fig. 4:**
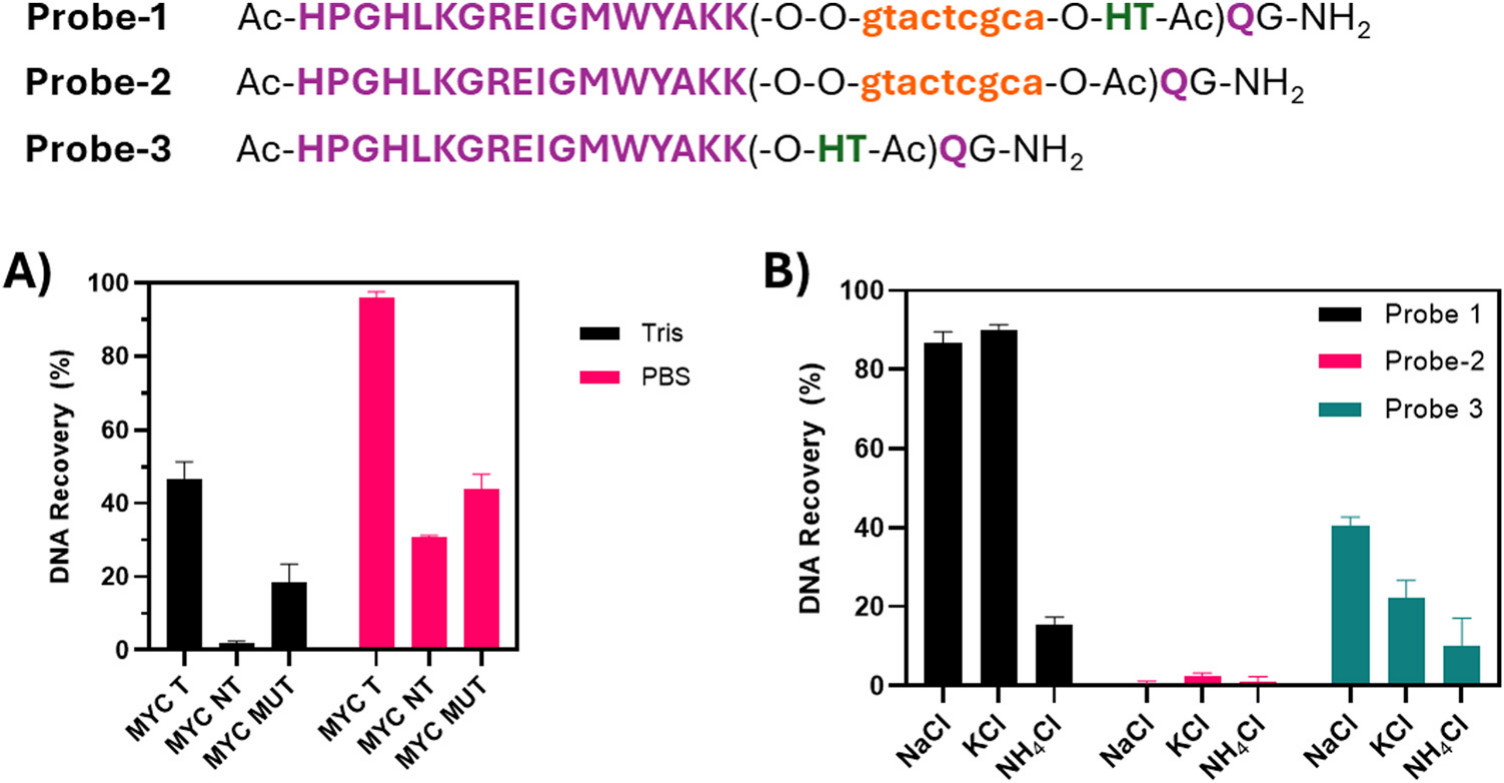
Optimization experiments performed varying (A) buffer (PBS 100 mM or tris, 10 mM, pH 7.8, 100 mM NaCl) and (B) salt composition (100 mM NaCl/KCl/or 10 mM NH_4_Cl in PBS 100 mM, pH 7.8). The experiments were performed at 5 μM strand concentration. Purple capitals indicate amino acids, orange letters PNA monomers; HT refers to His-Tag.

In the first preliminary experiment, various buffers, including phosphate-buffer saline (PBS, 100 mM) and tris(hydroxymethyl)aminomethane (tris HCl, 10 mM), at pH 7.8 and 100 mM of NaCl, both normally used as buffers that allow G4-folding, were used for the pull-down experiments. In this preliminary set-up, we performed the elution using the imidazole-containing elution buffer, as described above. For the purpose, we used Probe-1, screening the three DNAs MycT, NT and Mut. The experiment, upon HPLC analysis and performed in technical duplicate, showed that the pull-down efficiency was influenced by the buffer composition (Fig. 4(A)). The amine in tris-buffer might interact with the Ni–NTA beads, causing the reduction of the metal cation.^45^ We thus choose PBS as the buffer for the following experiments and kept a concentration of 5 μM for both the DNA sequences and Probe-1.

Next, we evaluated the ion type and concentration, crucial for the pull-down experiments (required to avoid non-specific interactions with the beads) and the folding of the G4 structure. From the literature, it is very well known that G4 structures are stabilized by monovalent cations, including, amongst others, sodium (Na^+^), potassium (K^+^) and ammonium (NH_4_^+^), following the stabilization pattern Na^+^< K^+^< NH_4_^+^. The three corresponding chloride (Cl^−^) salts were tested in 100 mM PBS buffer at 100 mM salt concentration. Ammonium chloride (NH_4_Cl) was also tested at a lower salt concentration (1 mM), given the high stabilization ability of the cation. Unfortunately, the experiments with NH_4_^+^ showed the lowest recovery (Fig. 4(B)), presumably due to interference with the Ni–NTA beads during the hybridization process. K^+^ and Na^+^ showed comparable results with high recovery for Probe-1, and for Probe-3 (Rhau peptide equipped with His-Tag, no PNA, used at 2.5 eq. with respect to the DNA to allow the formation of a complex with the target).^38^ As expected, no pull-down for Probe-2 (no pull-down tag) was recorded. Given the similar results obtained with the two salts with Probe 1, we arbitrarily decided to perform the following experiments using NaCl.

### Selectivity evaluation

Under the conditions described in the previous paragraph (PBS 100 mM, pH 7.4, 100 mM NaCl, at 5 μM strand concentration) we evaluated the pull-down efficiency for each of the synthesized probes (with the exclusion of Probe-2 (no His-Tag), which was used as a control in the initial phase of the optimization). We performed two different types of experiments: single target experiments (where only one DNA species was added to the mixture, and competition experiments, where two competing strands, MycT and NT, were added to the reaction mixture. This was done to evaluate eventual selectivity issues towards a similar G4-structure and the different HPLC retention times of the two species allowed for separate integration of the DNAs.

**Fig. 5:**
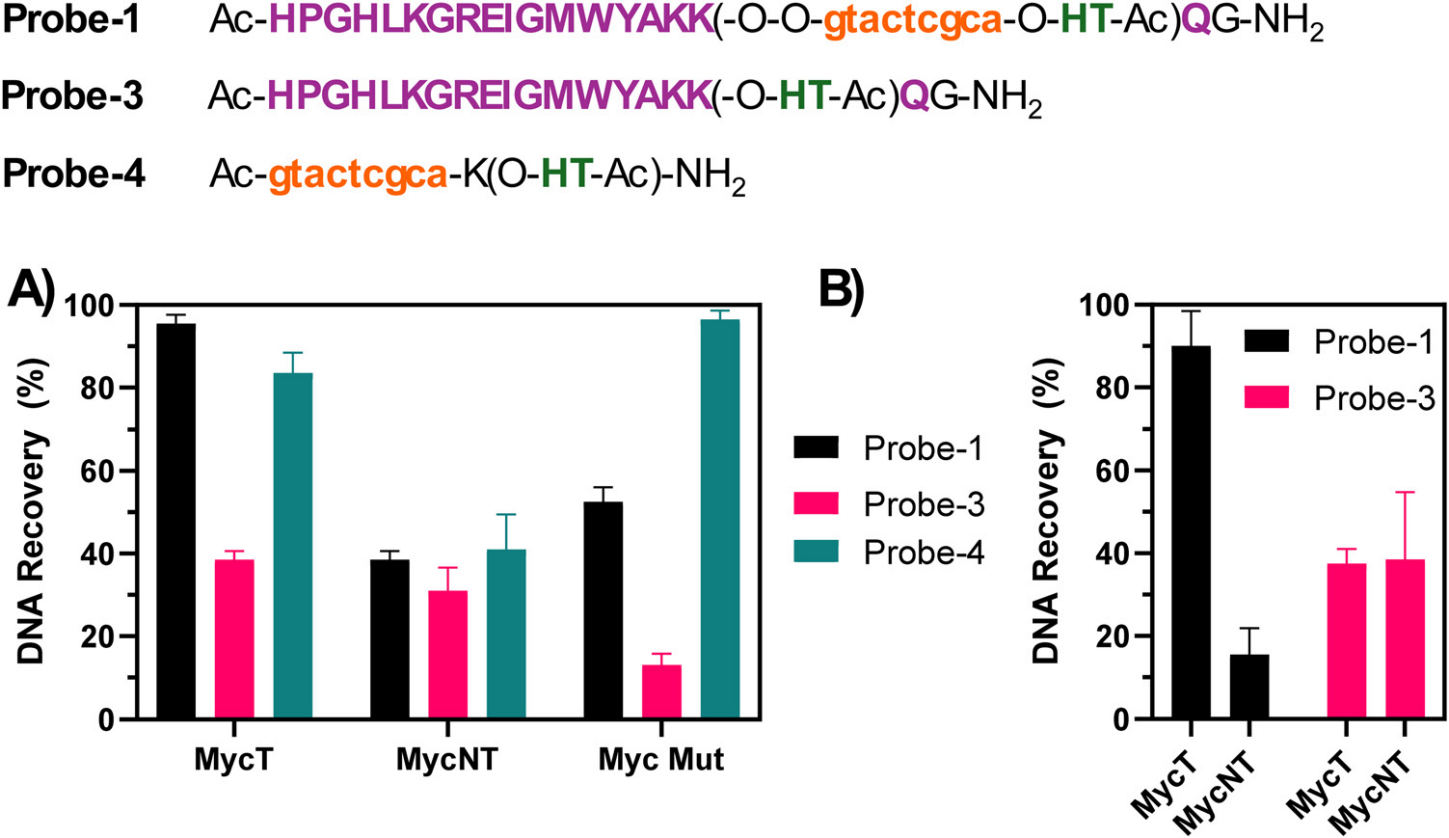
Pull-down selectivity evaluation for single target (A) and competition experiments (B). The experiments were performed at 5 μM strand concentration in 100 mM PBS buffer (100 mM NaCl, pH 7.8). For the experiments performed with Probe-3, 2.5 eq. of peptide (with respect to the DNA strand) were used.

The single target experiment (Fig. 5(A)) showed almost quantitative recovery of the targeted MycT sequence with Probe-1, very high recovery for Probe-4 (no G4-ligand), and a lower recovery (<40%) when using Probe-3 (no PNA). This lower pull-down for Probe-3 might reflect the lower stability of the Rhau peptide–DNA complex compared to the more stable PNA:DNA duplex. We believe that the micromolar *K*_d_ value of the DNA:RHAU complex,^38^ together with the modification of the lysine lateral side chain in position 17 (not essential for the binding, but still partly contributing to the binding of the G4 target through electrostatic interactions with the negatively charged DNA backbone), might contribute to reducing DNA–peptide complex formation. This, in turn could lead to a loss of target from the probe during the washing procedures and thus a lower recovery. A similar value of pull-down efficiency is seen when using Probe-1 in presence of MycNT, able to fold into a G4 but without a matching region needed for the PNA-hybridization. As expected, also in this case Probe-3 (no PNA) was able to pull down the sequence to a certain extent due to the Rhau peptide binding the G4-part of MycNT. Notably, recovery of the mutated MycMut sequence was lower when using Probe-1 as compared to Probe-4 (no G4-ligand). We attribute this phenomenon to the presence of a bulky ligand (Rhau) that has negative effects on the hybridization of the PNA to the target DNA sequence.

In contrast, when looking at the competition experiment where the two DNA sequences MycT and MycNT compete for the same probe (Fig. 5(B)), the selectivity of Probe-1 is enhanced due to the synergetic effects of the peptide ligand binding to the G4-structure and the PNA hybridizing to the DNA to form a duplex. Expectedly, Probe-3 did not exhibit significant preference for either target or non-target sequence, given the lack of sequence-specific recognition.

### Quantification on a 96-well plate format

#### G4–hemin DNAzyme formation: optimization

Next, we set out to evaluate the possibility of quantifying the DNA obtained from the pull-down experiments using the enzyme-like properties of the G4 structure, for a fast, virtually ‘in-line’ determination of the target concentration. Various conditions were optimized, including the concentration of H_2_O_2_, the substrate preparation (tetramethylbenzidine, TMB) and the DNA concentration range. Fresh TMB solution was preferred to avoid oxidation issues, and commercially available substrate solution (from ELISA kits) was not optimal due to high H_2_O_2_ concentration, causing noticeable substrate oxidation even in absence of DNA. The commercially available kit at our disposal (Thermo Scientific™ 1-Step™ TMB ELISA Substrate the ready-to-use formulation), already contains H_2_O_2_. Therefore, we believe that in this context, the traditional formulations where TMB substrate and H_2_O_2_ are separated and mixed only prior to the experiment, are more suitable. The optimal concentration of H_2_O_2_ to be used for the experiments was 0.05% (material and methods section, *vide infra*) and the concentration range of the experiment 1000–80 nM.

After the initial optimization, a calibration curve was generated using progressively diluted DNA solutions and performing the experiment in duplicate. In parallel, a pull-down experiment under the same conditions found in the previous paragraph (selectivity evaluation), was performed.

**Fig. 6:**
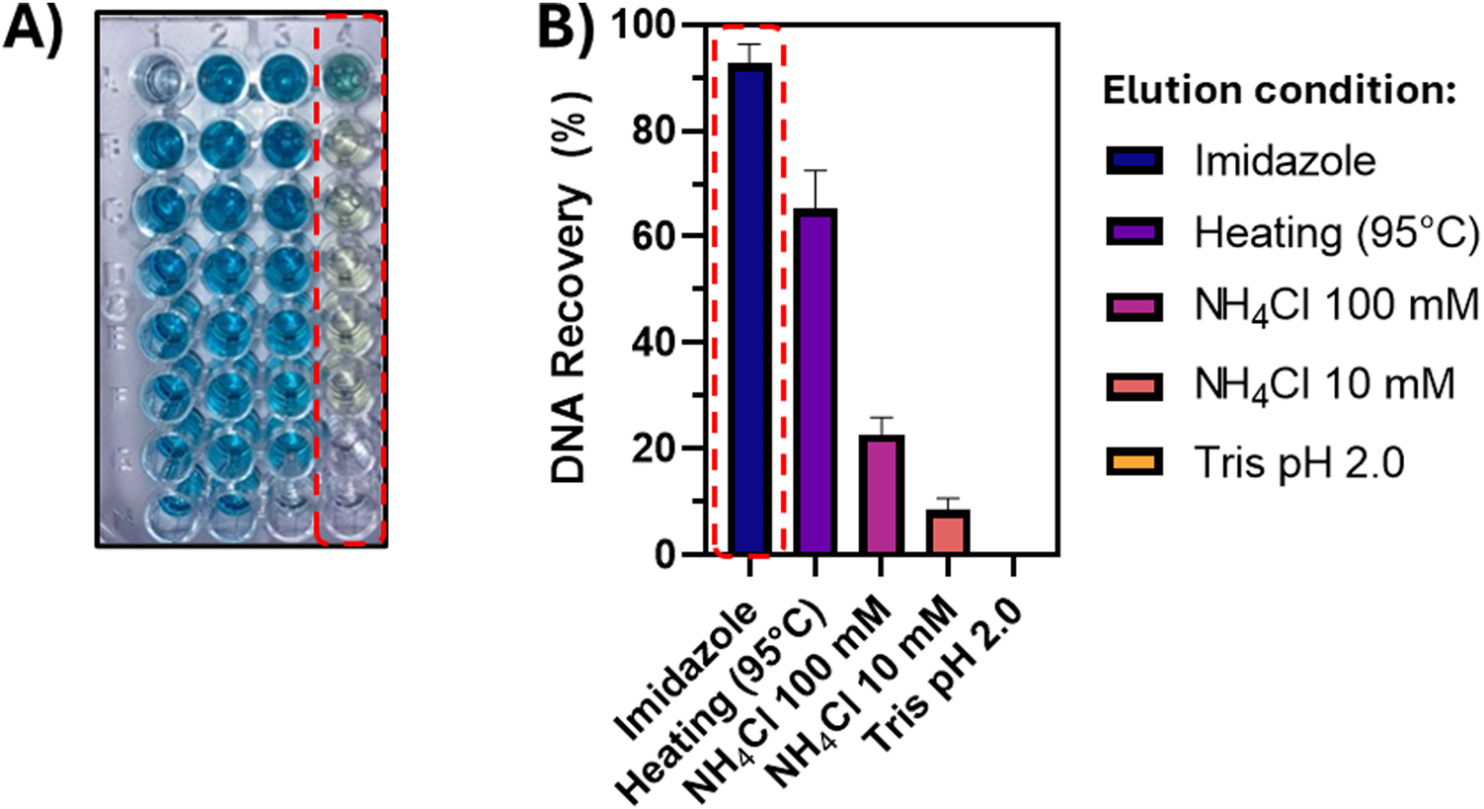
Optimization of the elution conditions. (A) Picture illustrating the result of TMB oxidation in presence of H_2_O_2_ and hemin–G4 complex, when using PBS without heating the sample (lane 1), after heating the sample (lane 2), in presence of NH_4_Cl 100 mM (lane 3) and in presence of imidazole (lane 4, circled in red). Progressively diluted solutions are included in the rows (A)–(H). (B) Resulting DNA recovery (as determined by HPLC) upon testing different elution conditions to try and avoid interference of imidazole elution buffer (B, red square). The experiment was performed at a fixed DNA concentration of 1 μM, at a final volume of 160 μL per well, using 20 μL of hemin (25 μM) and 40 μL of freshly prepared TMB (100 μM).

When adding the solution coming from the pull-down experiment to the 96-well plate containing the solution of TMB and hemin, a light-yellow color rather than the usual blue color of the oxidized TMB was observed upon addition of the H_2_O_2_ solution, which obviated quantification of the nucleic acid content of the solution (Fig. 6(A), lane 4). We believe that the imidazole, used in the elution buffer for the Ni–NTA magnetic beads, can either interfere with the oxidation step (acting as a mild reducing agent) or compete with the G4-DNA in solution for binding to the hemin, needed for the DNAzyme activity of the G4. To address this issue, we performed an additional experiment, using Probe-1 as pull-down probe, and screening different elution conditions as alternative to the imidazole elution buffer. Next to the addition of NH_4_Cl, Tris HCl at acidic pH (to interfere with the Ni–NTA ligand–His interaction), we also attempted heating the sample to 95 °C to melt the DNA:PNA complex and disrupt the Ni–NTA interactions.

Among the alternative conditions tested (Fig. 6(B)), sample heating seemed to represent the next best alternative to the originally used imidazole elution buffer. For the final 96-well plate experiment, we therefore decided to perform the heating of the sample to allow for the elution of the DNA from the beads.

### 96-well plate detection

**Fig. 7:**
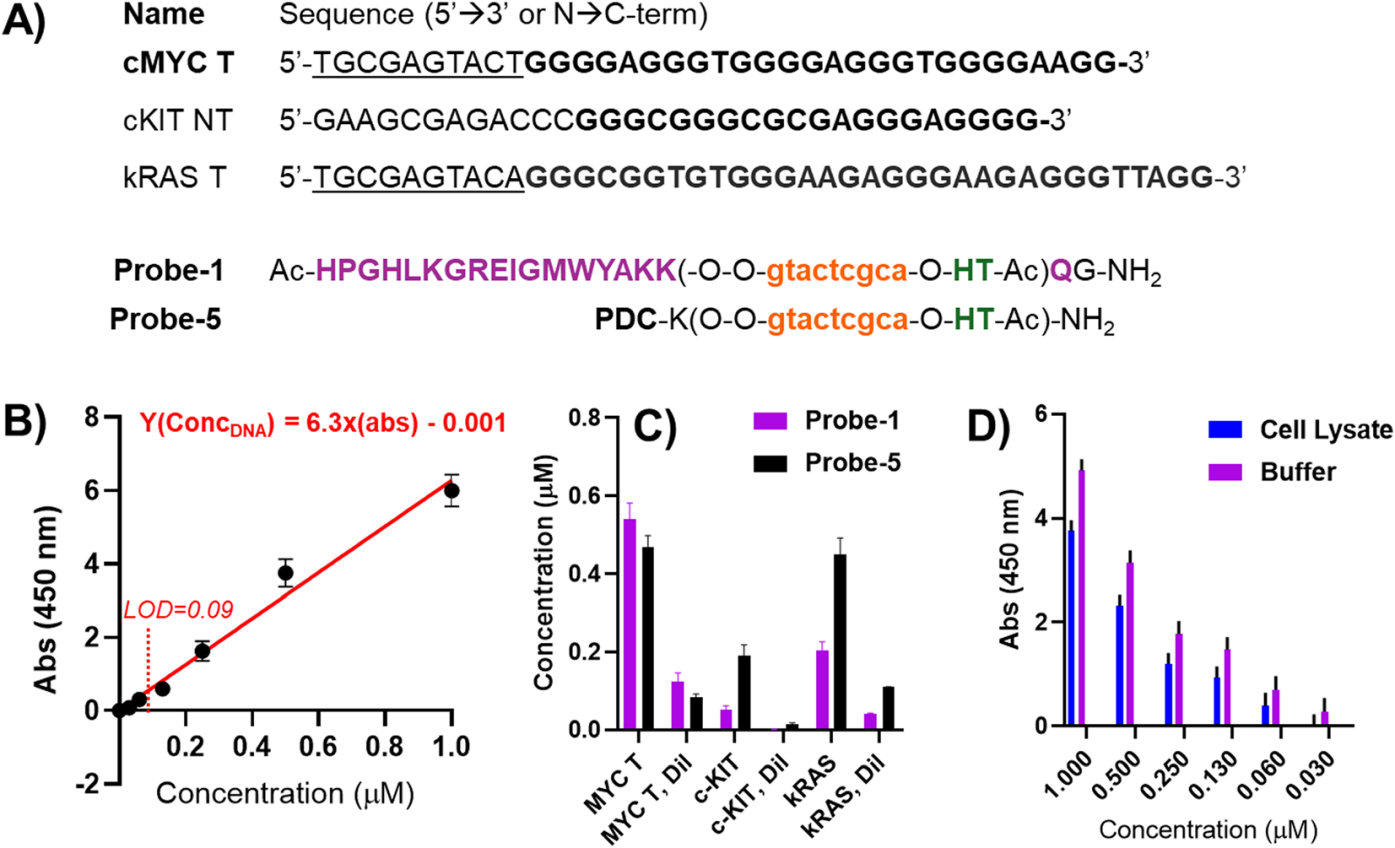
Set-up for the final pull-down experiment performed in a 96-well plate format. (A) Sequences of the target DNA sequences and probes used in the experiment; (B) calibration curve built using the MycT DNA sequence; (C) results of the pull-down experiment, after elution and quantification through plate reader, following the absorbance of the TMB solution at 450 nm wavelength. The pull-down experiment was performed at 5 μM strand concentration in 100 mM PBS buffer (containing 100 mM NaCl, pH 7.8). The 96-well plate quantification was performed at a final volume of 160 μL per well, using 20 μL of hemin (25 μM) and 40 μL of freshly prepared TMB (100 μM). LOD of the assay was 0.09 μM. (D) Comparative pull-down experiment between PBS buffer (purple bars) and cell lysate (CL, blue bars), in presence of increasing concentrations of cMYC T and a PNA Probe-1 concentration of 5 μM.

As a final experiment and upon defining the optimal elution conditions, we decided to use other alternative target and non-target sequences, containing each time sequences able to fold into a parallel G4, including cKIT (equipped with a non-matching flanking region) and kRAS (equipped with a matching flanking region to the probes used for the study) G4s (Fig. 7(A)). We compared the efficiency of Probe-1 (containing Rhau) and Probe-5 (containing the small-molecule PDC) (see Fig. 3 for the structures). Calibration curves (example in Fig. 7(B)) were cross-validated by evaluating pulled-down sample concentration at 1 : 1 and 1 : 4 dilutions, to check if the intensity of the signal would drop by the same factor.

In general (Fig. 7(B)), for both Probe-1 and Probe-5, we found good reproducibility (low standard deviation among the replicates) and linearity (to a 1 : 4 reduction of the sample corresponds a 1 : 4 reduction of the signal intensity). More in detail, for Probe-1, we found a higher signal for the fully-matched target MycT DNA, *versus* a low signal for the scrambled cKit NT DNA. The obtained result is expected in view of the presence of a mismatched flanking region (that does not allow for binding of the PNA) and the presence of a long loop on the top tetrad of the G4-structure in the target, that might sterically hinder Rhau from interacting with the structure, reducing the binding affinity in this setup. On the other hand, treating kRAS T DNA, containing the correct flanking region for PNA recognition, with Probe-1 resulted in a lower recovery in comparison to MycT DNA, but higher as compared to cKIT. A possible explanation for the lower recovery can be found in the binding mode of the peptide to the top tetrad of the structure, with the long loop sterically impeding the PNA:DNA duplex formation, and *vice versa*. In other words, the two binding events (PNA:DNA duplex and Rhau:G4 stacking) negatively influence each other, resulting in reduced binding to the DNA.

When using Probe-5, the recovery of cKIT, albeit lower than recovery of cMYC and kRAS, increases as compared to the Rhau-equipped Probe-1, while the pull-down of KRAS appears to be at the same level as the one of cMYC DNA. This result can be attributed to the higher promiscuity of the small-molecule binder, that can more easily adapt on the targeted structure, increasing the pull-down efficiency.

In a final experiment, we performed a pull-down of Myc-T DNA from cell lysate to compare two media (lysate and PBS buffer) and verify the recovery of the target in more relevant conditions for pull-down applications using a 96-well plate format. The cell lysate was obtained from the MDA-MB-231 cell line using a lysis buffer containing Triton X-100 and SDS, which could potentially interfere with PNA–DNA hybridization and reduce the efficiency of the pull-down. For practical reasons, we maintained the same PNA probe design (PNA Probe-1, complementary to Myc-T) and spiked the cell lysate with increasing amounts of the target (Fig. 7(D)). Although a global reduction in signal was observed compared to the same experiment performed in PBS buffer, the probe retained its ability to efficiently pull down the target without further assay optimization, showing only a minor reduction in detection sensitivity.

## Discussion

Many biomarker sequences of interest exhibit a G4-folding portion that can be conveniently exploited as tool to improve DNA isolation. The presence of the G4-handle further enables enzyme-free quantification of the isolated material that only requires a 96-well plate reader and cheap materials. This was achieved by exploiting the enzyme-like features of the G4:Hemin complex, which shows a behaviour typical of peroxidase enzymes. While the presented method relies on the presence of a folded G4 handle on the desired target, there is literature evidence of a consistent fraction of G4-folding sequences among pathological biomarkers that can be targeted.^31^ Hence, approaches dedicated to a simplified isolation and quantification of relevant G4-folding biomarkers can be considered as a valuable addition to the state-of-the-art.

In this work, we optimised the pull-down of DNA sequences in a step-by-step fashion and further demonstrated a simple and convenient method for their quantification. The latter is possible in a 96-well plate-based fashion thanks to the G4-folding portion of the isolated DNA, using TMB as a substrate and exploiting the DNAzyme properties of the target. We further showed that alternative tags, such as the HisTag that is commonly used for recombinant protein purification, can be used in place of the biotin-tag, generally used in the context of streptavidin-mediated DNA and RNA pull-down. In our hands, the Ni–NTA beads (developed for His-Tagged substrates) performed better when compared to SAv beads (developed for biotinylated substrates), enabling almost quantitative pull-down. As biotin is often endogenously present and could affect the assay for future applications in biological samples, the choice of the His-Tag, an unnatural tag that is generally inserted in recombinant proteins, rather than the more classically used (for pull-down purposed) biotin tag, offers considerable advantages. In addition to this, the Ni–NTA tag beads are cheaper compared to the SAv ones, which require protein immobilisation on the magnetic nanoparticle.

Pull-down experiments of more complex sequences, containing a G4-forming tract downstream of the targeted sequence, showed how the presence of a G4-ligand can increase the efficiency while maintaining a certain selectivity for the target. In principle, this could be optimized upon exploitation of more selective and tighter binders, such as the previously described ligands PDS, PhenDC3, BRACO-19, NDIs and other small molecules. In fact when moving to the 96-well plate format, the experiment with the alternative PDC-equipped probe rather than the Rhau-peptide equipped PNA, shows that other ligands can be used for the purpose of achieving pull-down. Furthermore, depending on the ligand used and the specificity of that ligand for the G4 structure, a higher degree of selectivity, on top of the sequence-selectivity ensured by the PNA part of the probe, can in principle be achieved. Among the factors that need to be taken into account, the use of a more promiscous ligand, able to bind to multiple different G4-forming structures, can lead to an increased pull-down efficiency at the expense of selectivity. While it was shown that the use of the wrong binder (*e.g.* use of Rhau for KIT quadruplex) can interfere with efficient recovery of a specific target sequence, entailing the need for an *ad hoc* optimization for specific cases, the here presented modular platform easily allows for that. Nevertheless, we also showed that is possible to use the quadruplex part of the target seqeunce as an extra selectivity handle. To conclude, even though the use of a G4-ligand on the probe is not mandatory for the experiment, it proves to be beneficial, as it can enhance selectivity over single-stranded (non G4-containing) sequences and positively influence the extent of DNA recovery.

For future applications, we foresee the translation of this method to RNA sequences containing G-rich tracts and able to fold into a stable G4, a property known for many biomarkers of interest such as miRNA, lncRNA and pre-miRNA, as well as certain mRNAs. The use of PNA–ligand conjugates to promote strand-invasion and induce G4-formation can be further used to quantify the sequence of interest, without the need for downstream enzymatic manipulations. We envisage the generation of small biosensors to detect nucleic acid biomarkers able to fold into quadruplexes structures *e.g.* for the pull-down of circulating RNA of interest with concurrent enzyme-free quantification.

## Material and methods

### Biotin-SAv pull-down

Prior to each experiment, Invitrogen™ Dynabeads™ MyOne™ C1 magnetic beads were vortexed for 30 seconds and transferred to a 1.5 mL Eppendorf tube. Following the manufacturer's protocol, beads were washed 3 times with 1× binding and washing buffer (Tris–HCl 10 mM, pH 7.8, 1 M NaCl, 0.5 mM EDTA) and resuspended in 30 μL of 2× binding and washing buffer. The beads were equilibrated with the sample for 30′, after which they were washed 5 times with 1 mL of binding and washing buffer and resuspended in 30 μL of Milli-Q water. The release of the DNA from the probes was achieved by incubating the beads at 95 °C for 10 minutes in an Eppendorf Thermomixer. The obtained solution was analysed by HPLC-UV.

### His-Tag pull-down

Prior to each experiment, DynaBeads™ His-Tag NTA Magnetic beads, were vortexed for 30 seconds and transferred to a 1.5 mL Eppendorf tube. Following the manufacturer's protocol, beads were washed 3 times with 1× binding and washing buffer (100 mM PBS, pH 8, 0.6 M NaCl, 0.02% Tween) and resuspended in 30 μL of 2× binding and washing buffer. The beads were equilibrated with the sample for 30′, after which they were washed 5 times with 1 mL of binding and washing buffer. The release of the DNA from the probes was achieved by incubating the beads with an elution buffer (PBS 50 mM + 300 mM NaCl) containing 0.3 M imidazole. The obtained solution was analysed by HPLC-UV.

### Modified His-Tag pull-down protocol

Prior to each experiment, HisPur™ Ni–NTA magnetic beads, were vortexed for 30 seconds and transferred to a 1.5 mL Eppendorf tube. The beads were washed 3 times with the modified binding and washing buffer (100 mM PBS, 100 mM NaCl) and resuspended in 30 μL of 2× binding and washing buffer. The beads were equilibrated with the sample for 30′, after which they were washed 5 times with 1 mL of binding and washing buffer and resuspended in 30 μL of Milli-Q water. The release of the DNA from the probes was achieved by incubating the beads at 95 °C for 10 minutes in an Eppendorf Thermomixer. The obtained solution was analysed by HPLC-UV or added to a 96-well plate for the TMB quantification experiments (*vide infra*).

### 96-well plate experiments

Prior to each experiment, a stock solution of TMB (100 μM) and hemin (25 μM, diluted from a stock of 1 mM in DMSO) was freshly prepared in mQ water. In each well, 20 μL of hemin solution were added. In a typical experiment, a 40 μL of progressive dilution of DNA (1000 nM, 500 nM, 0.25 nM, 0.125 nM, 0.06 and 0.03 nM, for the final volume of 160 μL in each well required for the analysis), was made to build a calibration curve. The samples containing the pulled-down DNA (40 μL) were subsequently added to the wells, and allowed to equilibrate at room temperature on an orbital shaker for 15′. Then, 40 μL of TMB substrate was added to each well, equilibrate for 5 minutes and, subsequently, 20 μL of an aqueous solution containing 0.05% H_2_O_2_ was also added. The plate was shaken at room temperature until a blue color appeared in solution. 40 μL of the stop solution (2 M sulfuric acid) was added, and the absorbance of the wells was measured in a plate reader (Trinean dropquant) for quantification. For the pull-down experiments from cell-lysate, a similar procedure was adopted: the buffer solution was substituted by a cell lysate obtained from a culture of MDA-MB-231 cells (10 million cells per ml), suspended in a phosphate lysis buffer (10 mM phosphate, pH 7.6) containing 0.01%SDS, 0.5% Triton-X, and 0.5% sodium deoxycholate, supplemented with 150 mM NaCl.

## Data availability

The data supporting this article has been included as part of the ESI.† The original raw data from HPLC chromatogram or NMR crude file can be requested to the corresponding authors.

## Conflicts of interest

There are no conflicts to declare.

## Supplementary Material

CB-OLF-D4CB00185K-s001
