# Supplementary material for: Development of a His-Tag-mediated pull-down and quantification assay for G-quadruplex containing DNA sequences
Source: RSC Chem Biol. 2024 Nov 28;6(1):56–64. doi: 10.1039/d4cb00185k (PMC11613956; doi:10.1039/d4cb00185k)
Supplement: CB-006-D4CB00185K-s001 [file CB-006-D4CB00185K-s001.pdf]

Supplementary Information (SI) for RSC Chemical Biology.
This journal is © The Royal Society of Chemistry 2024

Development of a His-Tag-mediated pull-down and quantification assay for G-Quadruplex containing DNA Sequences

Enrico Cadoni*, Hanne Moerman, Annemieke Madder*

Organic and Biomimetic Chemistry Research Group, Department of Organic and Macromolecular Chemistry, Ghent University, Krijgslaan 281-S4, 9000 Gent, Belgium

Email: Annemieke.madder@ugent.be ; Enrico.cadoni@ugent.be

General

All reagents were purchased from Sigma-Aldrich, Fluka, Merck, TCI Europe, Fluorochem and used without further purification. Dry DMF was stored over 4 Å molecular sieves. TLCs were run on Merck silica 60 on aluminum sheets. Column chromatography was performed as flash chromatography on Grace silica 60 (0.060- 0.200 mm). DNA sequences were purchased from IDT (Leuven, Belgium).

HPLC-MS data were collected on an Agilent 1100 Series instrument equipped with a Phenomenex Kinetex C18 100 Å column (150 x 4.6 mm, 5 µm at 35 °C) connected to an ESMSD type VL mass detector (quadrupole ion trap mass spectrometer) with a flow rate of 1.5 mL/min was used with the following solvent systems: (A): 0.1% HCOOH in H_2O and (B) MeCN. Gradient: 100% A for 2 min, then a gradient from 0 to 100% B over 6 min was used, followed by 2 min of flushing with 100% B (further referred to as HPLC1 conditions) or 100% A for 0.5 min, a gradient from 0 to 10% B over 0.1 min and then from 10 % to 30 % B over 7.7 minutes was used, followed by 2 min of flushing with 100% B (further referred to as HPLC1 conditions).

HPLC-UV data were collected on an Agilent 1100 Series instrument equipped with a Waters X-Bridge BEH C18 XP Column (130Å, 2.5 µm, 4.6 mm X 50 mm) connected to a DAD using a flow rate of 0.80 ml/min with the following solvent system: (A) 0.1 M TEAA-buffer + 5% MeCN and (B) MeCN. Column was flushed for 4 minutes with (A), then a gradient from 0 to 100% B in 12 minutes was used, followed by 4 minutes flush of 100% B, and 4 minutes flush of 100% A (HPLC2 Conditions); PNA oligomers were purified using a Luna C18(2) (5 µm, 100 Å, 250x10 mm) (further referred to as HPLC3 conditions: 100% A for 5 min, then a gradient from 0 to 50% B over 30 min at a flow rate of 4.0 mL/min)

UV-Vis measurements (PNA quantification) were performed on a Thermo Scientific Nanodrop instrument, measuring the absorption at 260nm. TMB quantifications on a 96well plate format were performed on a Trinean Dropquant plate reader.

Ligand synthesis

The synthesis of the 306A ligand (PDC-COOH, **Figure S1**) was carried out according to previous literature reports.[1]

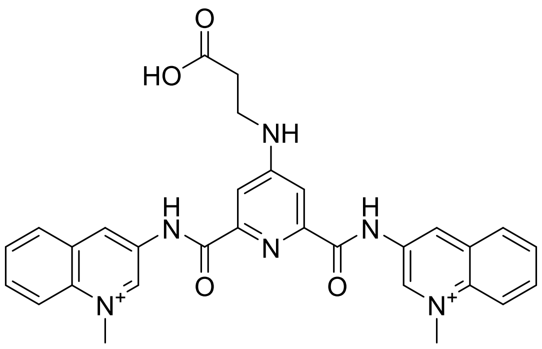

Figure 1. Structure of PDC-COOH introduced in Probe-5.

Probe Synthesis

The synthesis of the PNA and peptide sequences used in this work was performed with standard machine-assisted (Intavis Peptide Services, Tubingen) Fmoc-based solid-phase synthesis using HBTU/DIPEA as coupling mixture and commercially available Fmoc-monomers (PolyOrg for PNA monomers, Iris Biotech for amino acids), on Rink-Amide Chem Matrix as resin (loading: 0.2 mmol/g). For the modification on the lysine side chain (for His-tag or ligand introduction), functionalizations were performed after Dde deprotection. For a 5 µmol scale, this was achieved by shaking the resin vigorously for 1h in a solution containing 250 mg hydroxylamine hydrochloride and 184 mg imidazole in 1.2 mL NMP/DMF 5:1). [2]

Table S1. Sequences of the probes used in this study. PNA nucleobases are written in lowercase, and aminoacid residues are written in uppercase. Ac = acetyl; HT = His-tag;o = amino ethoxy-ethoxy acetyl spacing unit.

Probe	Sequence (N→C term)	MW	Yield %
PNA-H1	Ac-taggtgaag-RRK(**HT**)-NH_2	3844.4	15.9
PNA-H2	Ac-taggtgaagga-RRK (**HT**) -NH_2	4410.7	14.3
PNA-H3	Ac-taggtgaaggaaa-RRK (**HT**) -NH_2	4961.4	10.0
PNA-B1	Ac- taggtgaag -RRK (**Biotin**) -NH_2	3247.9	12.1
PNA-B2	Ac- taggtgaagga -RRK (**Biotin**) -NH_2	3814.4	11.4
PNA-B3	Ac- taggtgaaggaaa -RRK (**Biotin**) -NH_2	4364.6	11.3
Probe-1	Ac-HPGHLKGREIGMWYAKK(-o-o-gtactcgca-o-**HT**-Ac)QG-NH_2	6227.8	10.3
Probe-2	Ac-HPGHLKGREIGMWYAKK(-o-o-gtactcgca-o-**Ac**)QG-NH_2	5129.4	4.0
Probe-3	Ac-HPGHLKGREIGMWYAKK(o-**HT**-Ac)QG-NH_2	3517.2	9.4
Probe-4	Ac-gtactcgca-K(o-**HT**-Ac)-NH_2	3889.6	10.2
Probe-5	PDC-K(o-o-gtactcgca-o-**HT**-Ac)-NH_2	4649.2	21.5

Probe Characterizations

PNA-B1

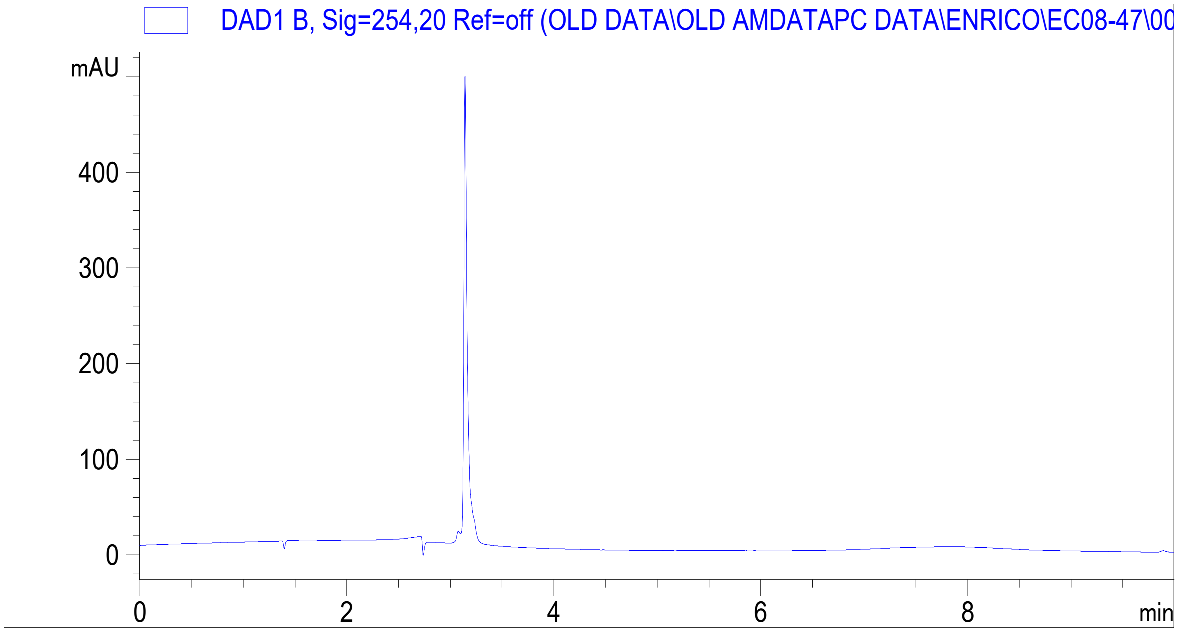

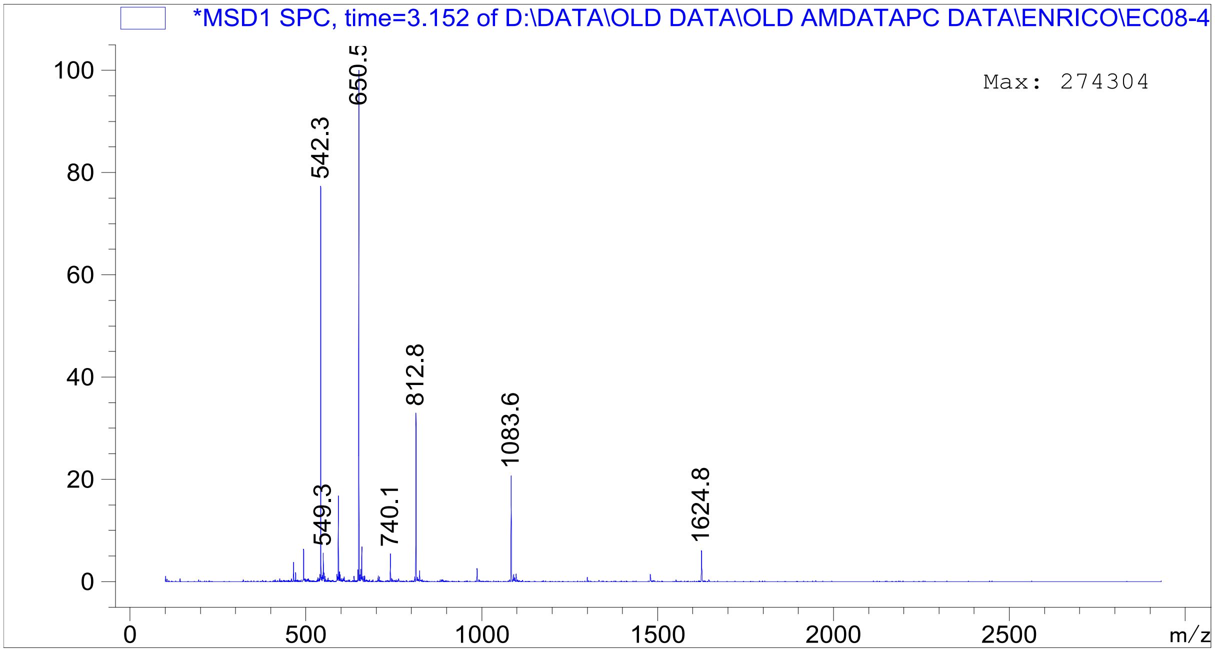

Figure S2. HPLC-MS chromatogram (HPLC1) of purified PNA-B1. HPLC-UV trace at 254 nm (top) and MS spectrum of the corresponding peak (bottom). Calcd MW: 3247.9, m/z found: 1624.8 $[M+2H]^{2+}$, 1083.6 $[M+3H]^{3+}$, 812.8 $[M+4H]^{4+}$, 650.5 $[M+5H]^{5+}$, 542.3 $[M+6H]^{6+}$.

PNA-B2

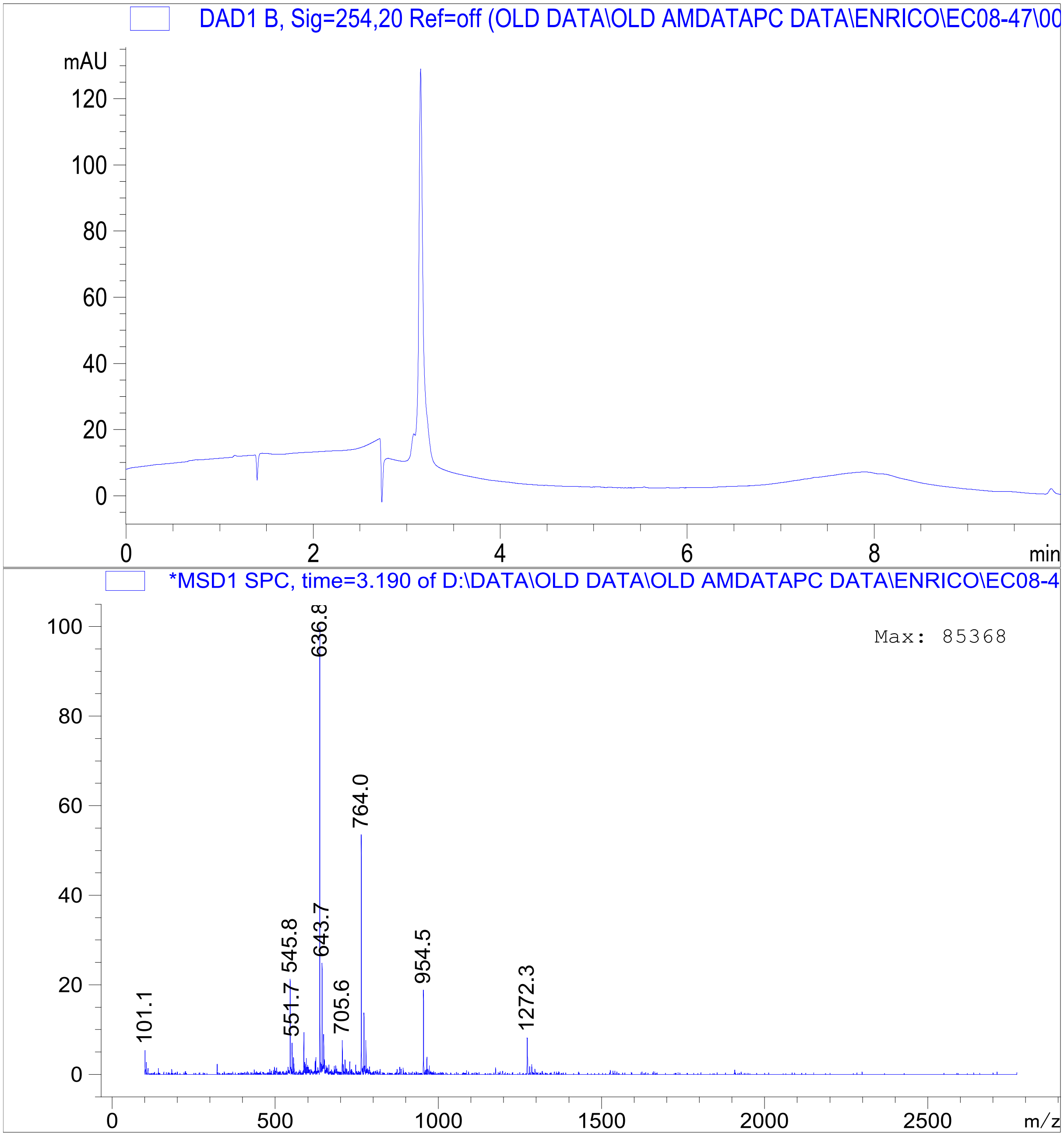

Figure S3. HPLC-MS chromatogram (HPLC1) of purified PNA-B2. HPLC-UV trace at 254 nm (top) and MS spectrum of the corresponding peak (bottom). Calcd MW: 3814.4, m/z found: 1272.3 $[M+3H]^{3+}$, 954.5 $[M+4H]^{4+}$, 764.0 $[M+5H]^{5+}$, 636.8 $[M+6H]^{6+}$.

PNA-B3

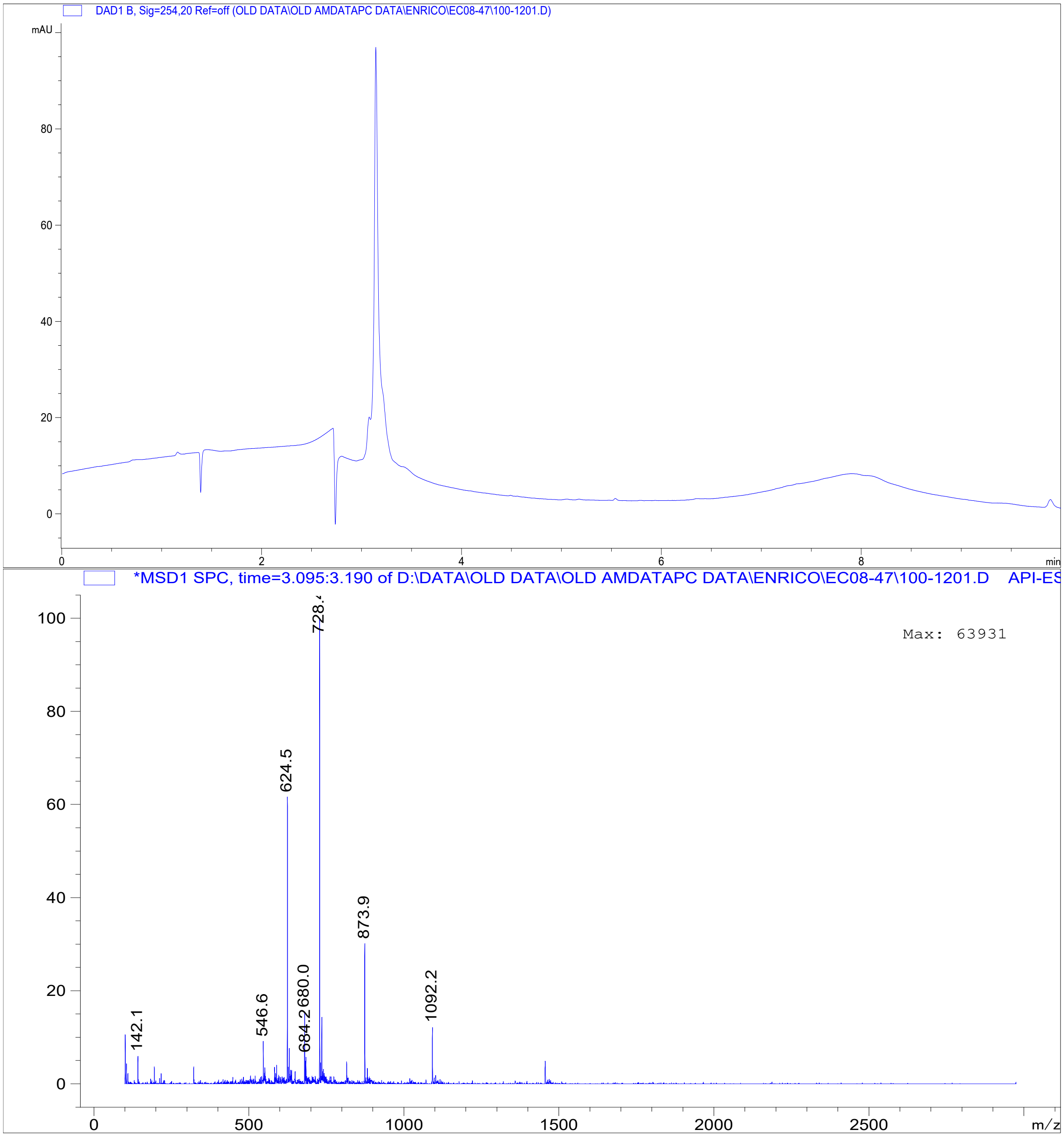

Figure S4. HPLC-MS chromatogram (HPLC1) of purified PNA-B3. HPLC-UV trace at 254 nm (top) and MS spectrum of the corresponding peak (bottom). Calcd MW: 4364.6, m/z found: 1092.2 $[M+4H]^{4+}$, 873.0 $[M+5H]^{5+}$, 728.4 $[M+6H]^{6+}$, 624.5 $[M+7H]^{7+}$.

PNA-H1

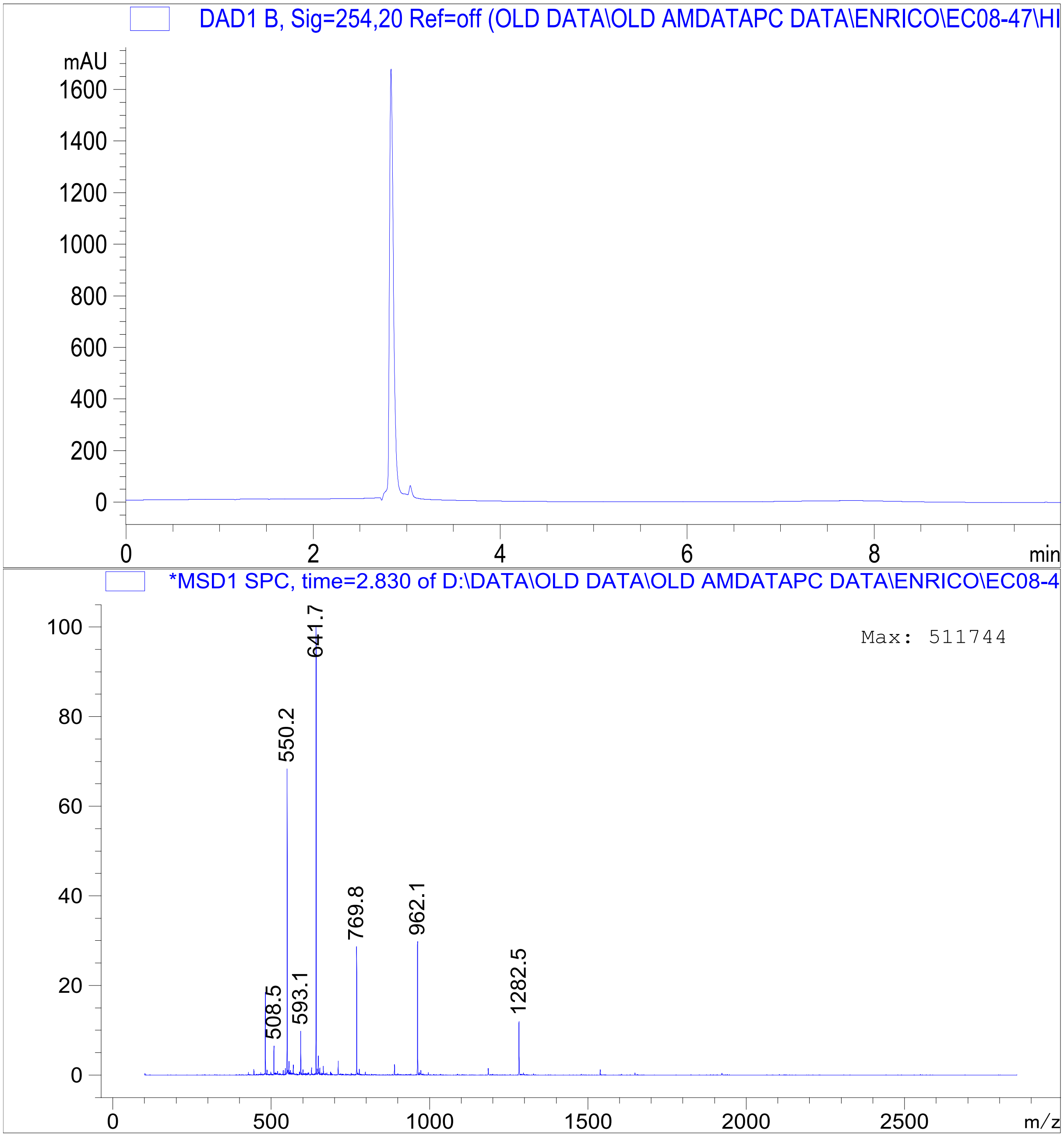

Figure S5. HPLC-MS chromatogram (HPLC1) of purified PNA-H1. HPLC-UV trace at 254 nm (top) and MS spectrum of the corresponding peak (bottom). Calcd MW: 3844.4, m/z found: 1282.5 $[M+3H]^{3+}$, 962.1 $[M+4H]^{4+}$, 769.8 $[M+5H]^{5+}$, 641.7 $[M+6H]^{6+}$, 550.2 $[M+7H]^{7+}$.

PNA-H2

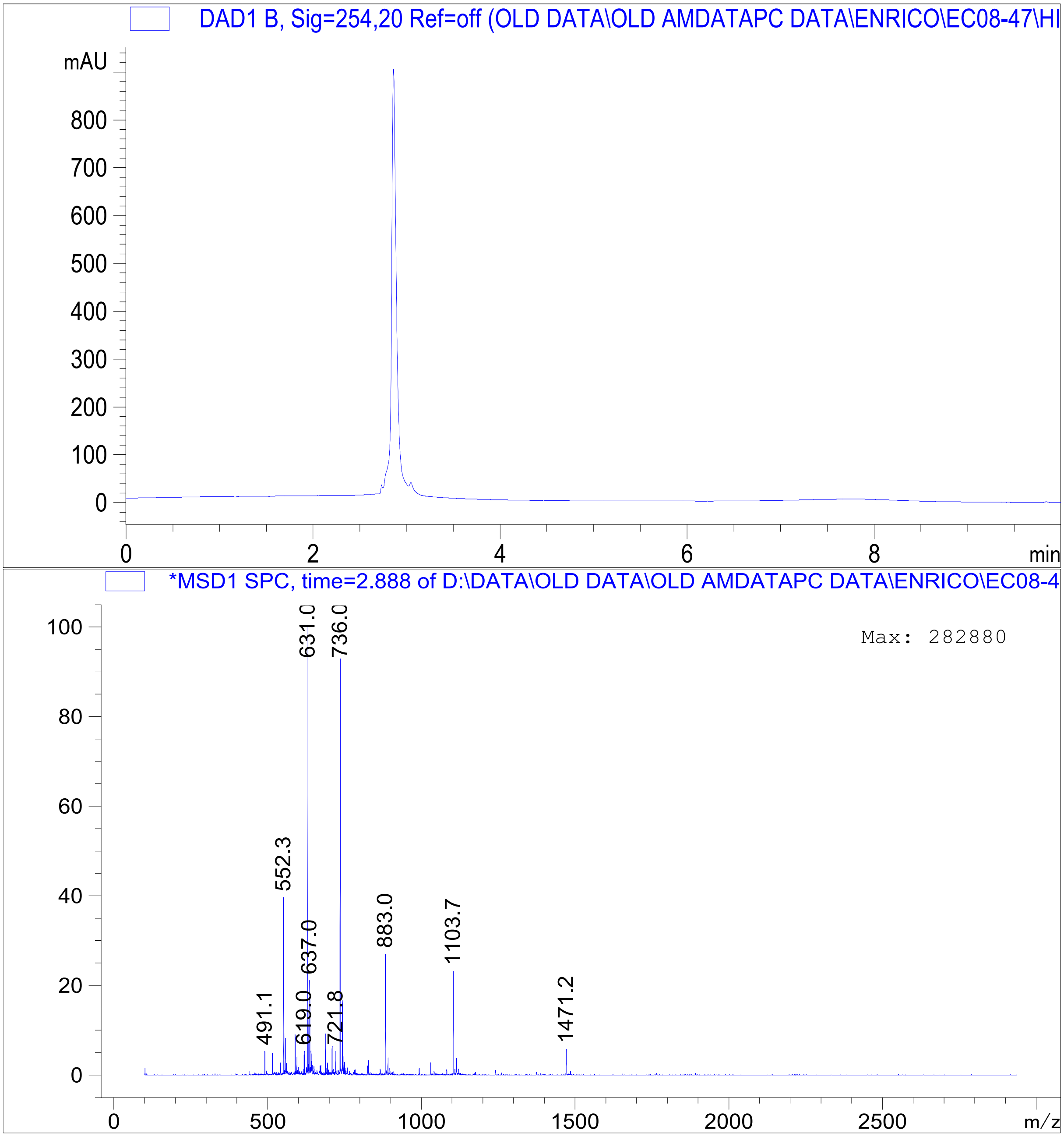

Figure S6. HPLC-MS chromatogram (HPLC1) of purified PNA-H2. HPLC-UV trace at 254 nm (top) and MS spectrum of the corresponding peak (bottom). Calcd MW: 4410.7, m/z found: 1471.2 $[M+3H]^{3+}$, 1103.7 $[M+4H]^{4+}$, 883.0 $[M+5H]^{5+}$, 736.0 $[M+6H]^{6+}$, 631.0 $[M+7H]^{7+}$.

PNA-H3

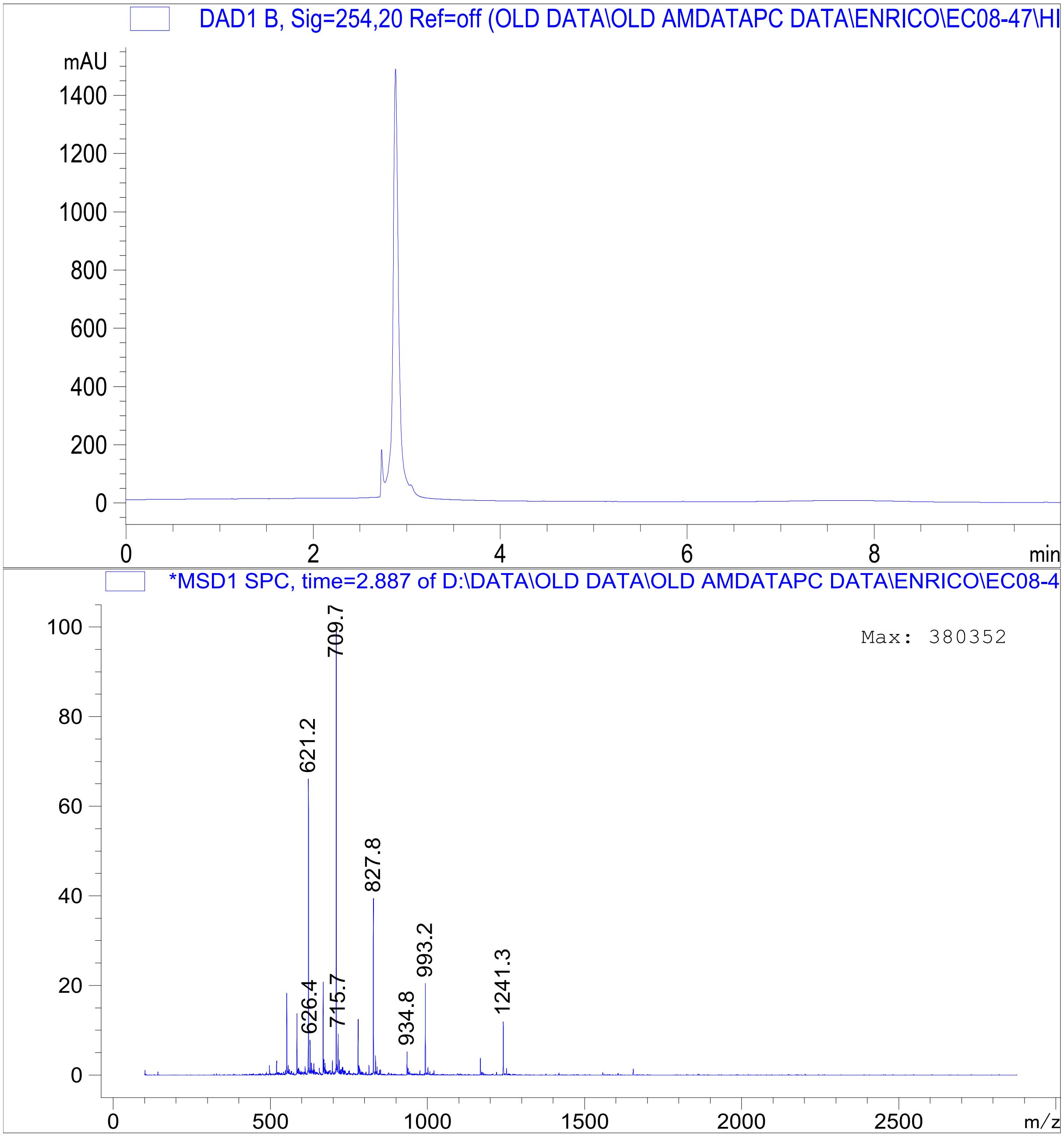

Figure S7. HPLC-MS chromatogram (HPLC1) of purified PNA-H3. HPLC-UV trace at 254 nm (top) and MS spectrum of the corresponding peak (bottom). Calcd MW: 4961.4, m/z found: 1241.3 $[M+4H]^{4+}$, 993.2 $[M+5H]^{5+}$, 827.8 $[M+6H]^{6+}$, 709.7 $[M+7H]^{7+}$, 621.2 $[M+8H]^{8+}$.

Probe-1

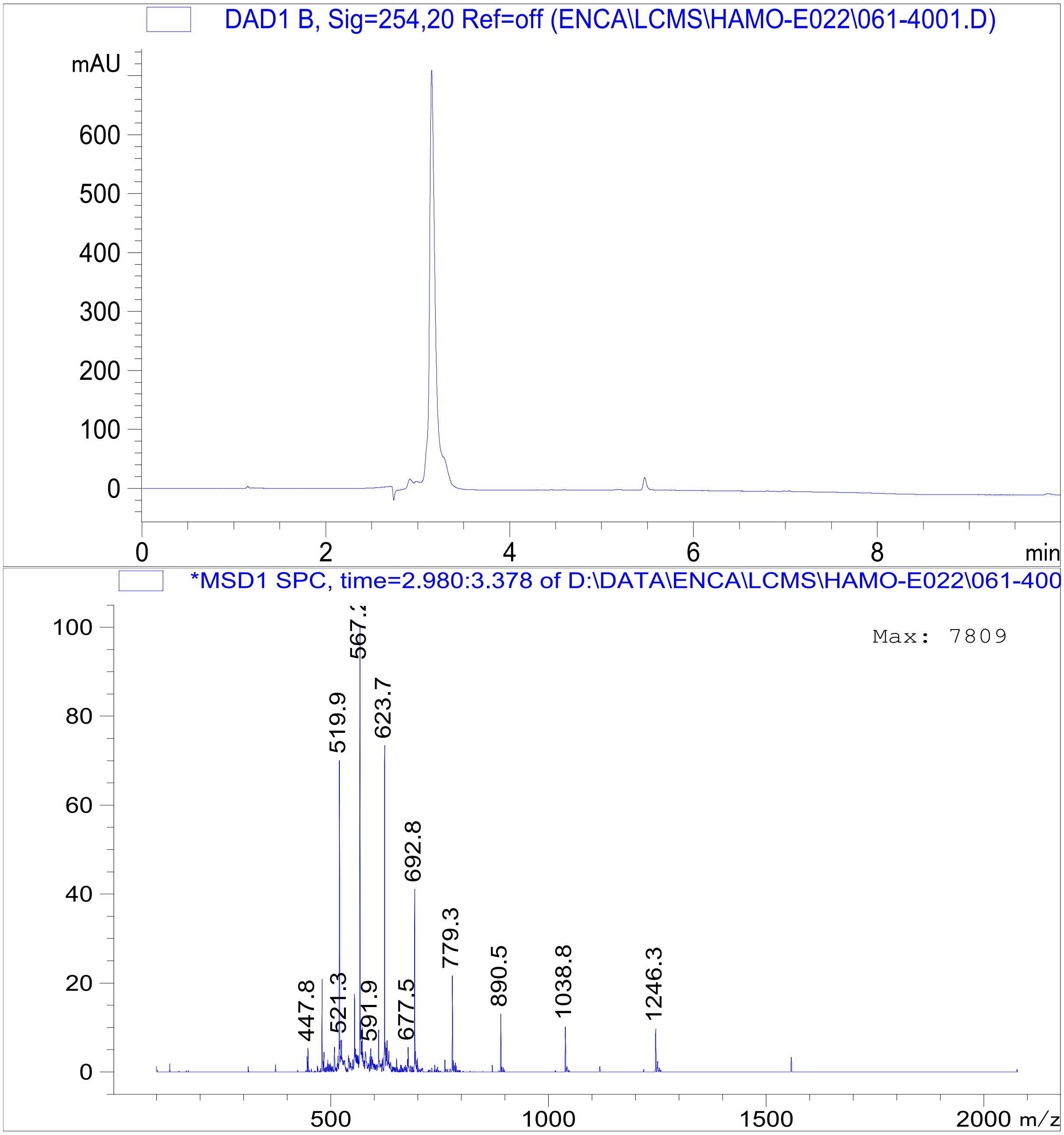

Figure S8. HPLC-MS chromatogram (HPLC1) of purified Probe-1. HPLC-UV trace at 254 nm (top) and MS spectrum of the corresponding peak (bottom). Calcd MW: 6227.8, m/z found: 1246.2 [M+5H]$^{5+}$, 1038.7 [M+6H]$^{6+}$, 890.5[M+7H]$^{7+}$, 779.3 [M+8H]$^{8+}$, 692.8 [M+9H]$^{9+}$, 623.7 [M+10H]$^{10+}$, 567.2 [M+11H]$^{11+}$, 519.9 [M+12H]$^{12+}$.

Probe-2

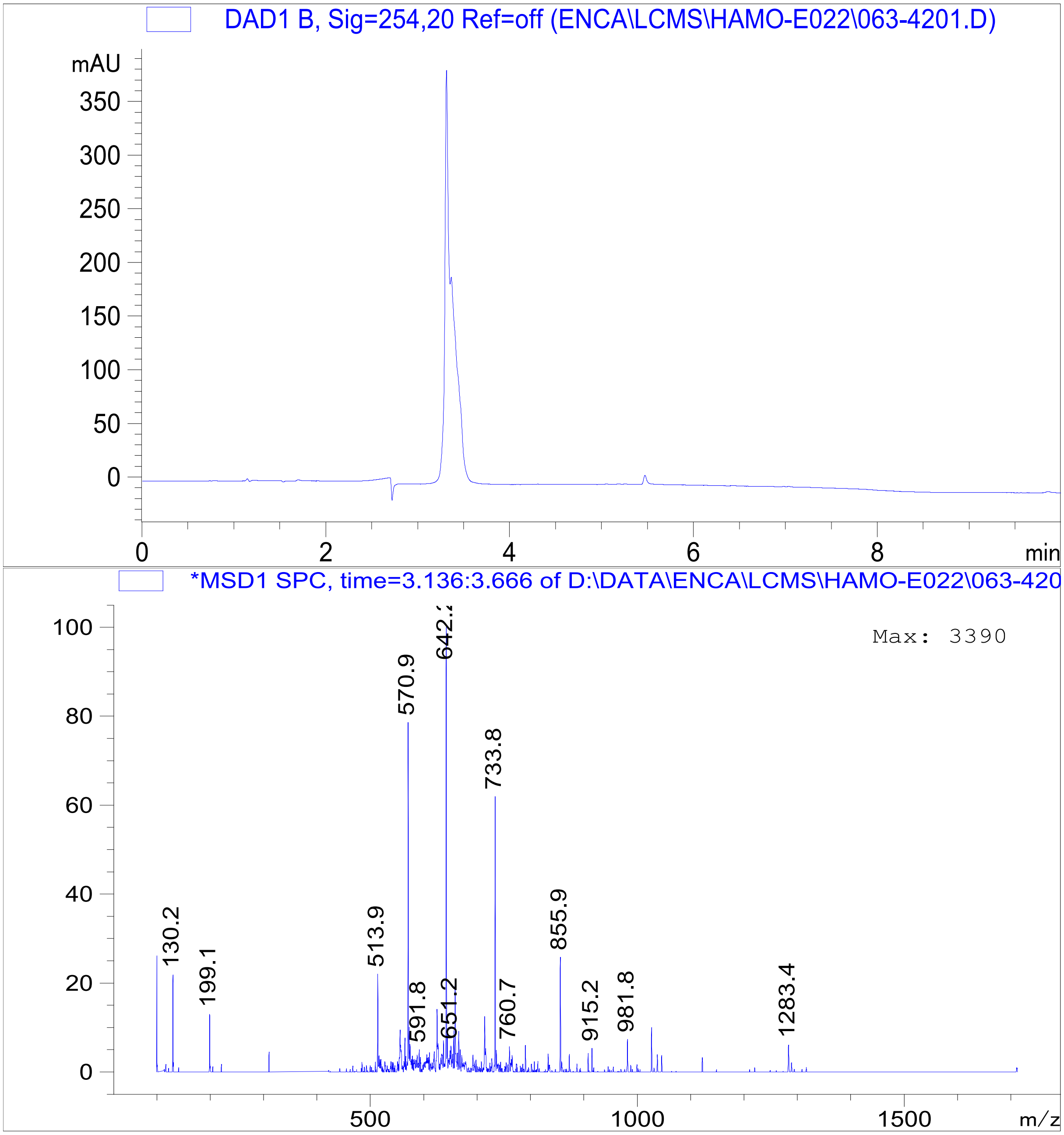

Figure S9. HPLC-MS chromatogram (HPLC1) of purified Probe-2. HPLC-UV trace at 254 nm (top) and MS spectrum of the corresponding peak (bottom). Calcd MW: 5126.4, m/z found: 1283.4 $[M+4H]^{4+}$,1025.2 $[M+5H]^{5+}$, 855.9 $[M+6H]^{6+}$, 733.8 $[M+7H]^{7+}$, 642.2 $[M+8H]^{8+}$, 570.9 $[M+9H]^{9+}$, 513.9 $[M+10H]^{10+}$.

Probe-3

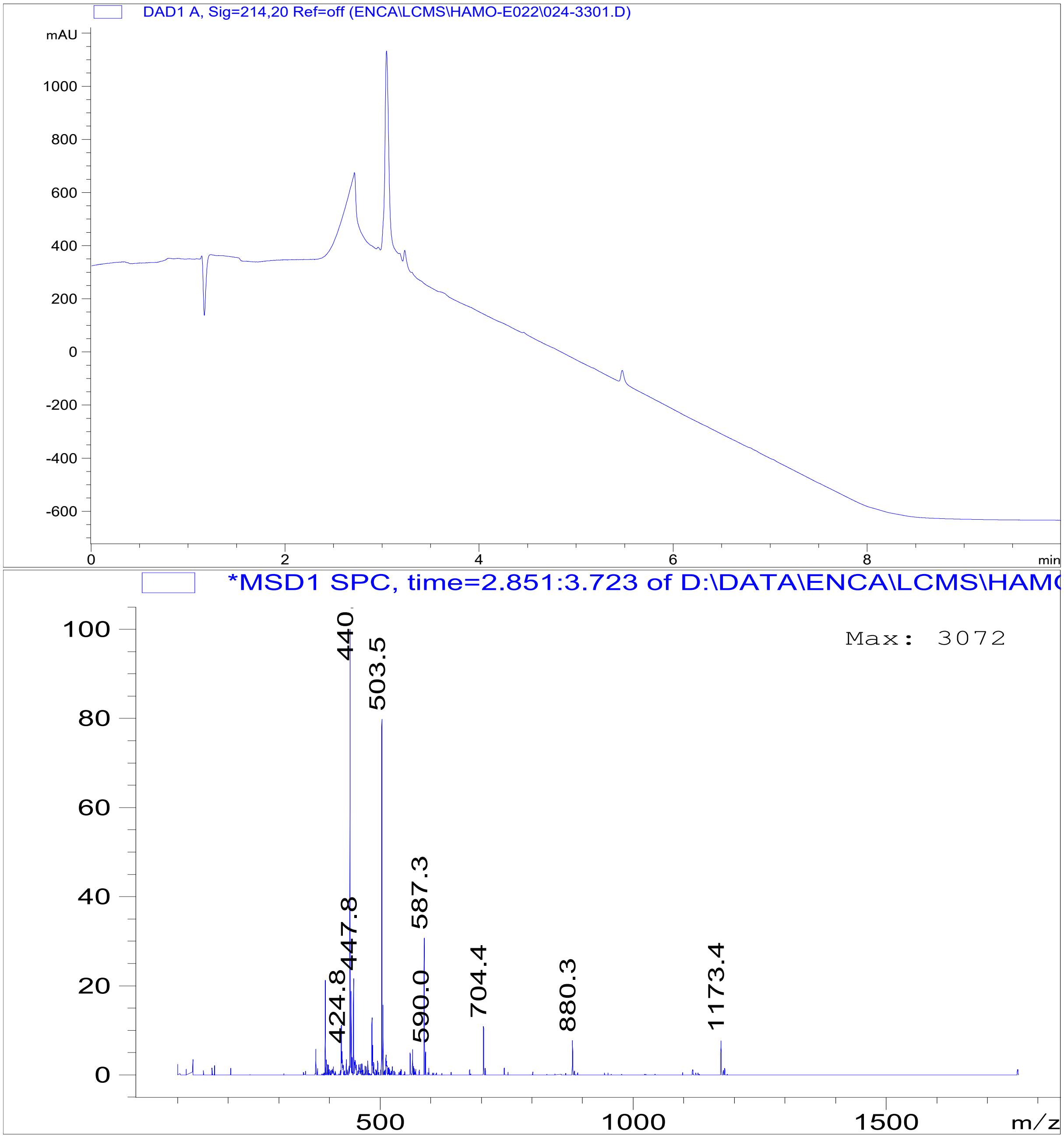

Figure S10. HPLC-MS chromatogram (HPLC1) of purified Probe-3. HPLC-UV trace at 214 nm (top) and MS spectrum of the corresponding peak (bottom). Calcd MW: 3518.2, m/z found: 1173.4 $[M+3H]^{3+}$, 880.3 $[M+4H]^{4+}$, 704.4 $[M+5H]^{5+}$, 587.3 $[M+6H]^{6+}$, 503.5 $[M+7H]^{7+}$, 440.7 $[M+8H]^{8+}$.

Probe-4

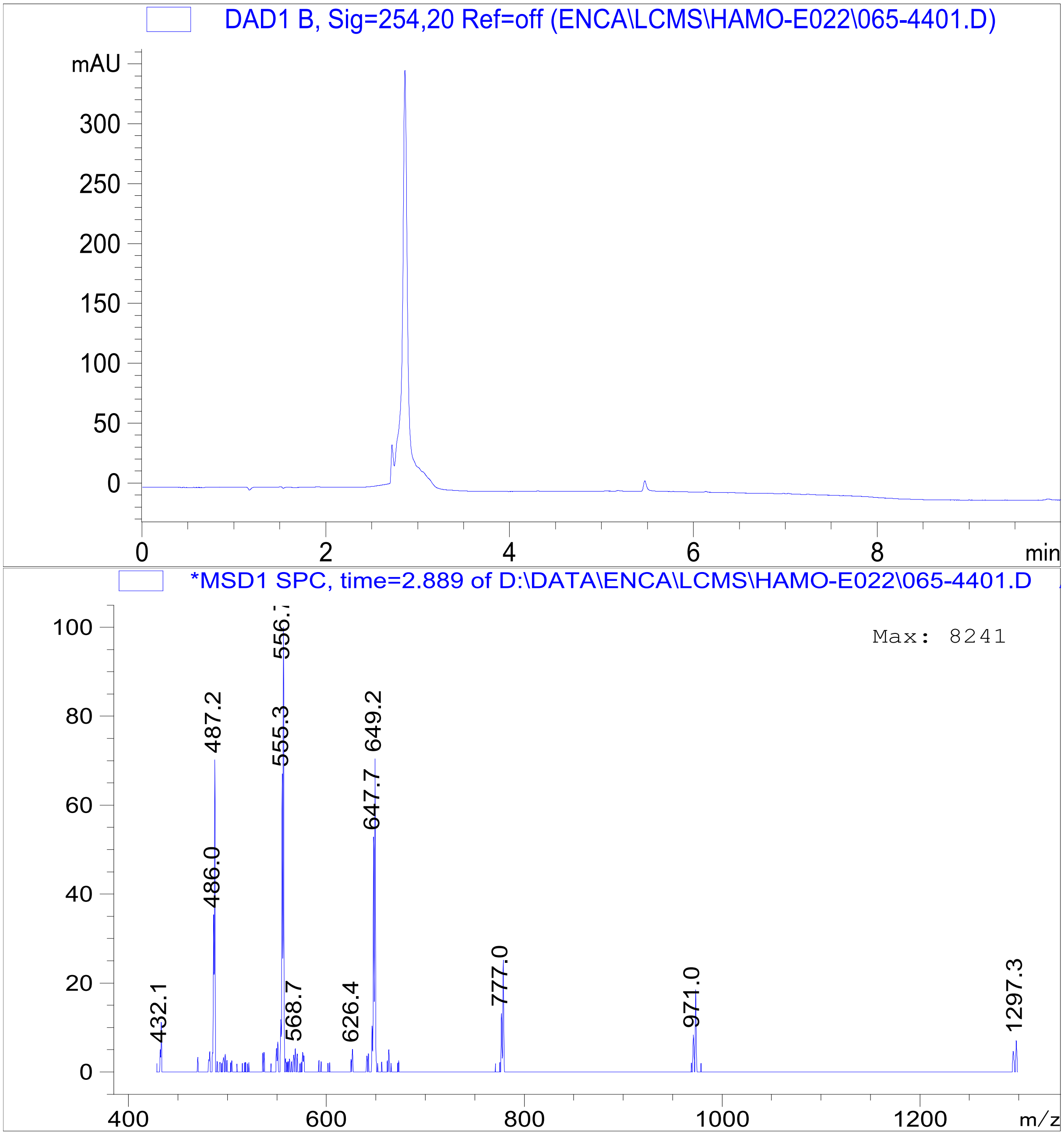

Figure S11. HPLC-MS chromatogram (HPLC1) of purified Probe-4. HPLC-UV trace at 254 nm (top) and MS spectrum of the corresponding peak (bottom). Calcd MW: 3886.6, m/z found: 1297.3 $[M+3H]^{3+}$,971.0 $[M+4H]^{4+}$,777.0 $[M+5H]^{5+}$, 647.7 $[M+6H]^{6+}$, 555.3 $[M+7H]^{7+}$, 487.2 $[M+8H]^{8+}$.

Probe-5

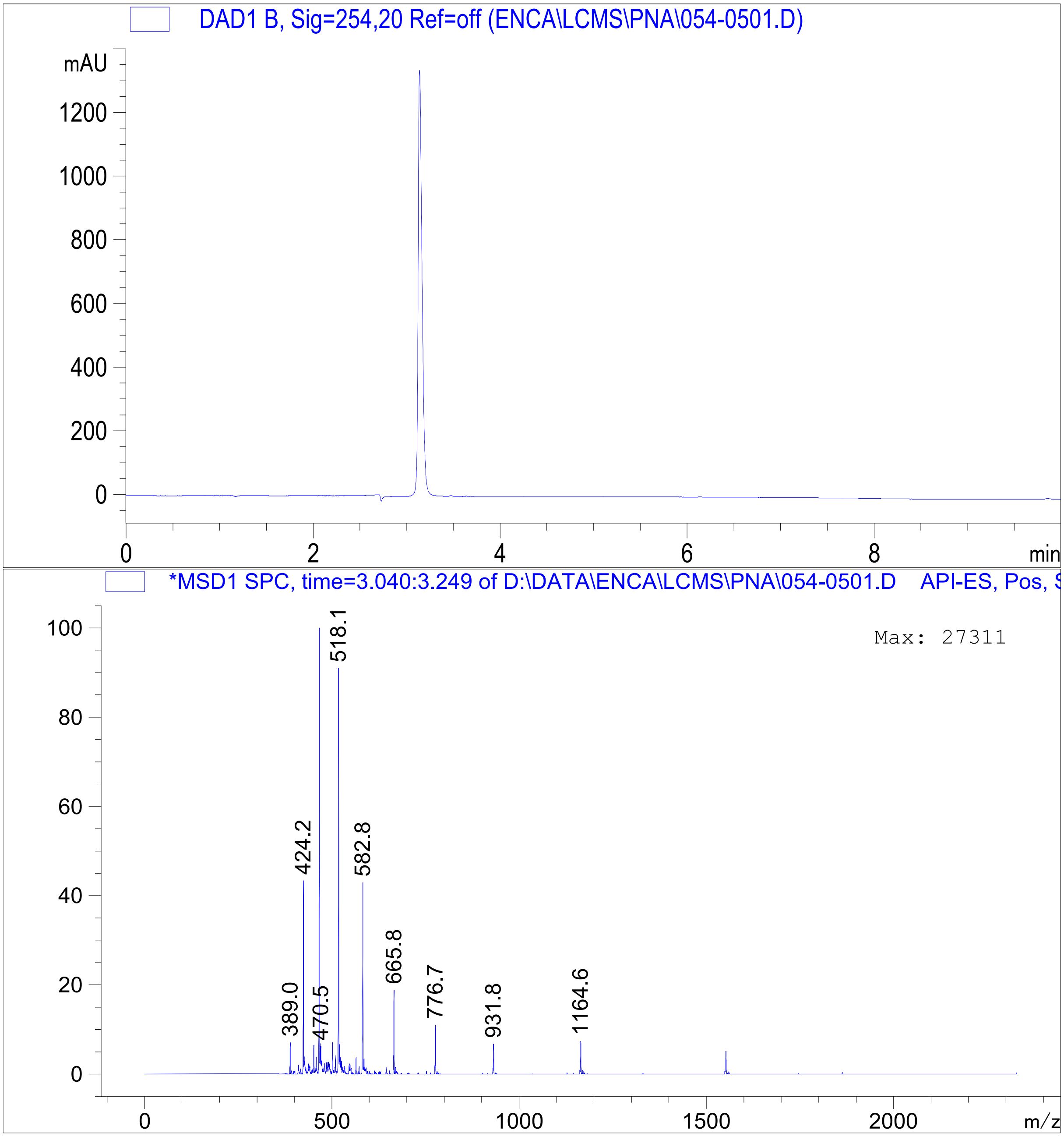

Figure S12. HPLC-MS chromatogram (HPLC1) of purified Probe-5. HPLC-UV trace at 254 nm (top) and MS spectrum of the corresponding peak (bottom). Calcd MW: 4649.2, m/z found: 1164.6 $[M+4H]^{4+}$,931.8 $[M+5H]^{5+}$, 776.7 $[M+6H]^{6+}$, 665.8 $[M+7H]^{7+}$, 582.8 $[M+8H]^{8+}$,518.1 $[M+9H]^{9+}$.

Pull-down experiments raw data

HPLC-UV recovery quantification

For the pull-down experiments analysed through HPLC-UV, each solution coming from the pull-down (please refer to the material and methods section in the main paper) was injected and the peak corresponding to the DNAs was integrated and normalized to a standard solution of the DNA target at 5 μM concentration.

Representative chromatograms from the pull-down experiments

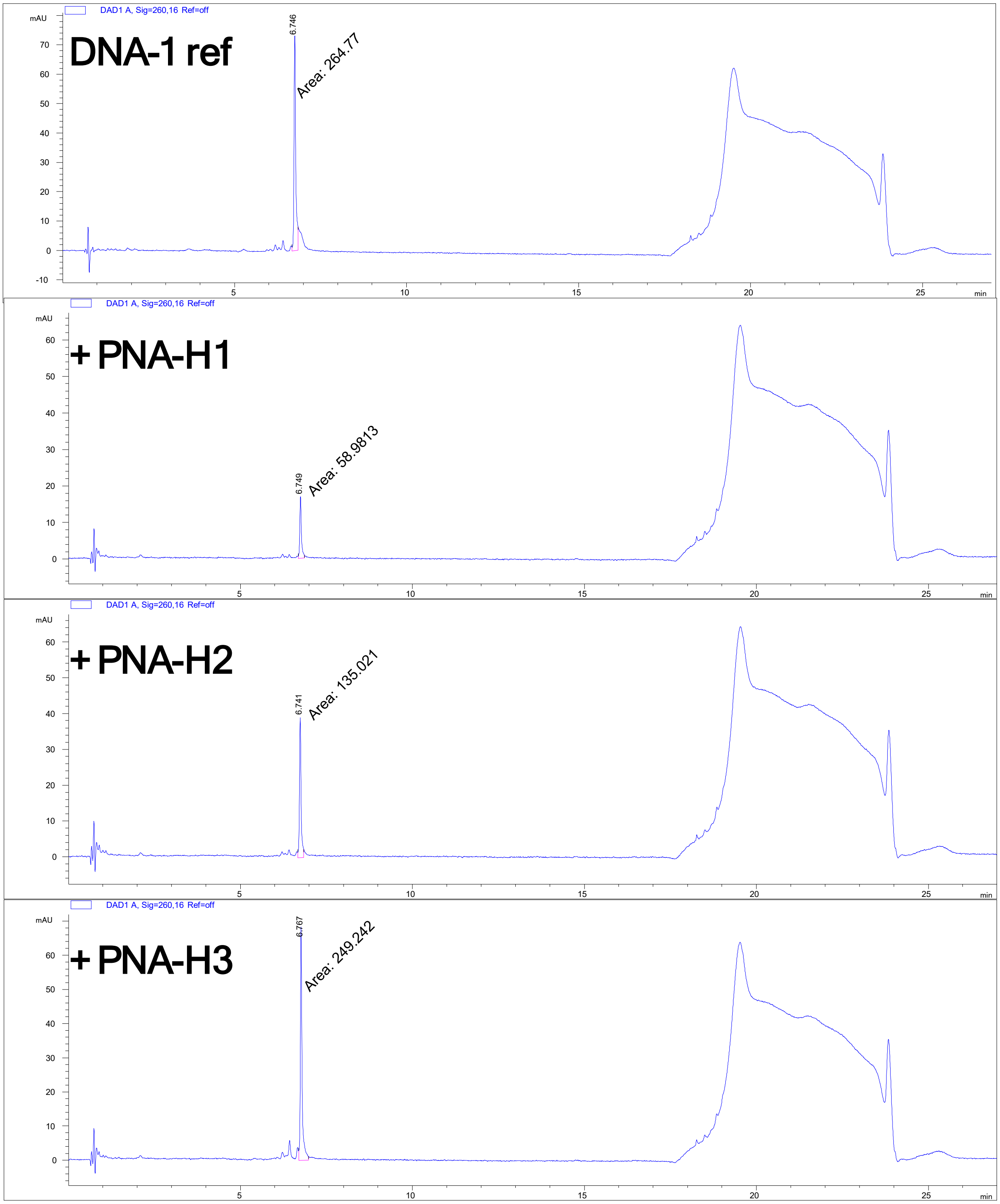

Figure S13. Pulldown experiment of DNA-1 in presence of PNA-H1, PNA-H2 and PNA-H3. The experiments were performed at 5 μM strand concentration in Tris-HCl buffer (0.1 M NaCl, pH 7.8).

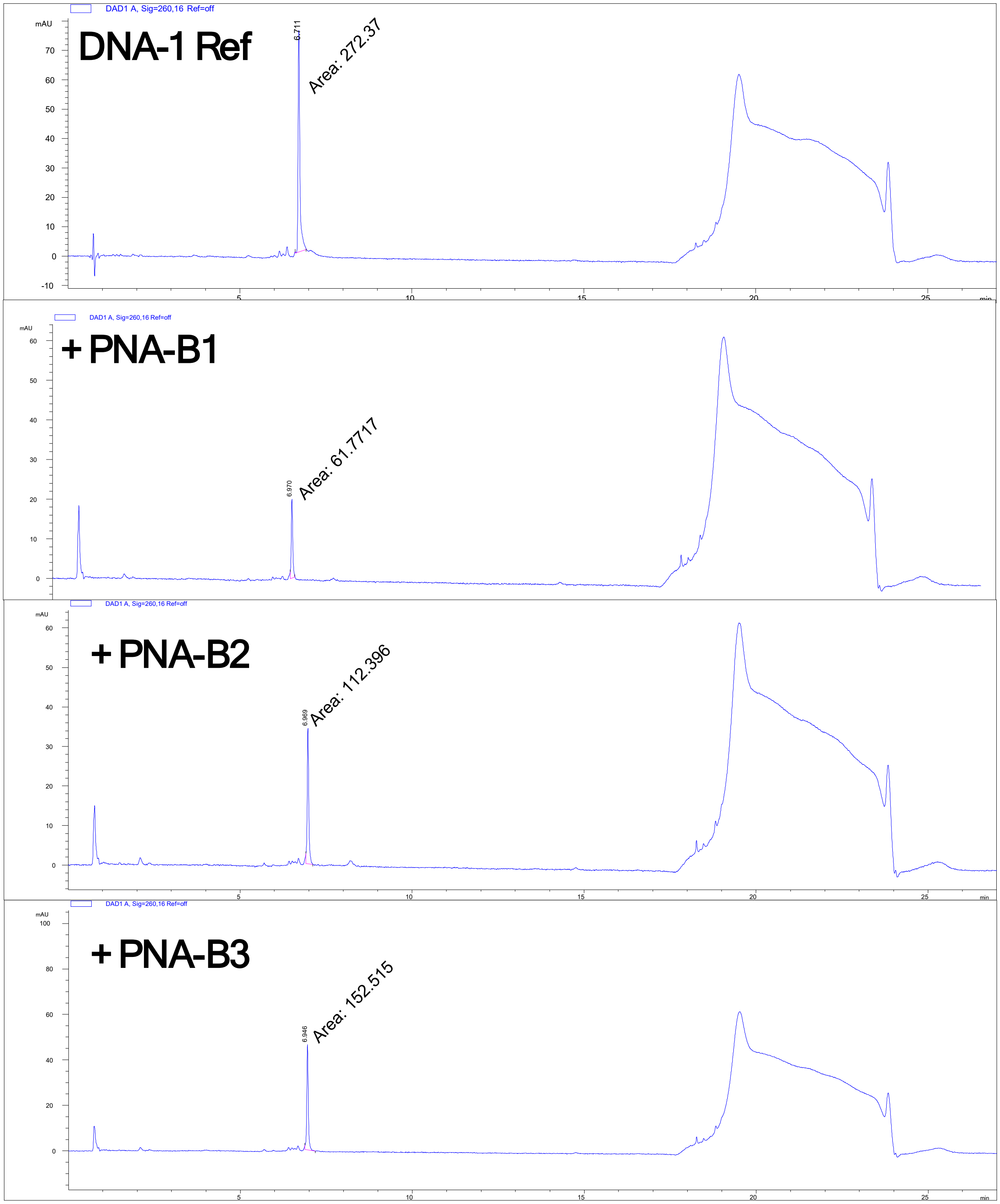

Figure S14. Pulldown experiment of DNA-1 in presence of PNA-B1, PNA-B2 and PNA-B3. The experiments were performed at 5 μM strand concentration in Tris-HCl buffer (0.1 M NaCl, pH 7.8).

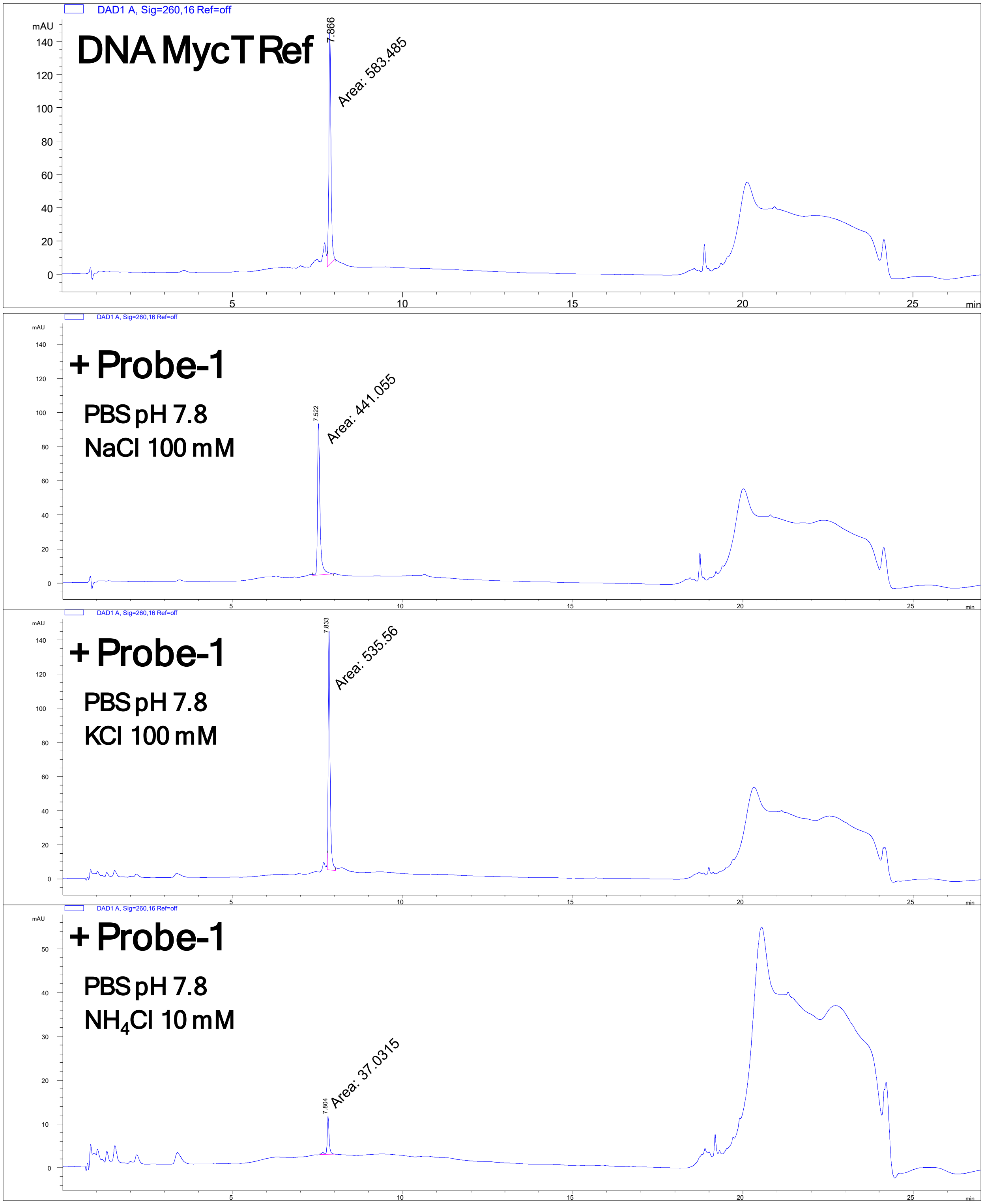

Figure S15. Pulldown experiment of DNA MycT in presence of Probe-1. The experiments were performed at 5 µM strand concentration in PBS buffer 0.1M (10-100 mM salt concentration, pH 7.8).

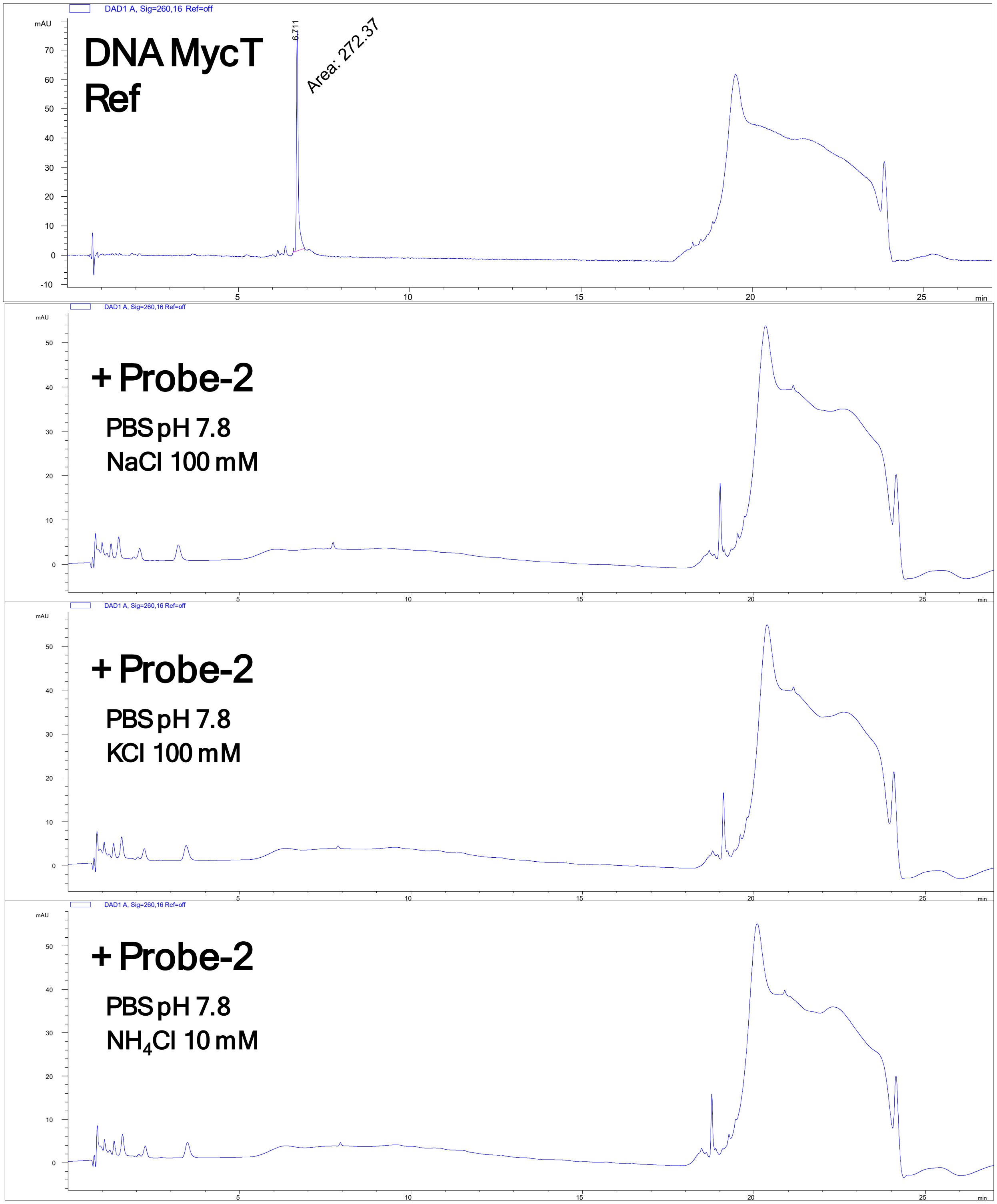

Figure S16. Pulldown experiment of DNA MycT in presence of Probe-2. The experiments were performed at 5 μM strand concentration in PBS buffer 0.1M (10-100 mM salt concentration, pH 7.8).

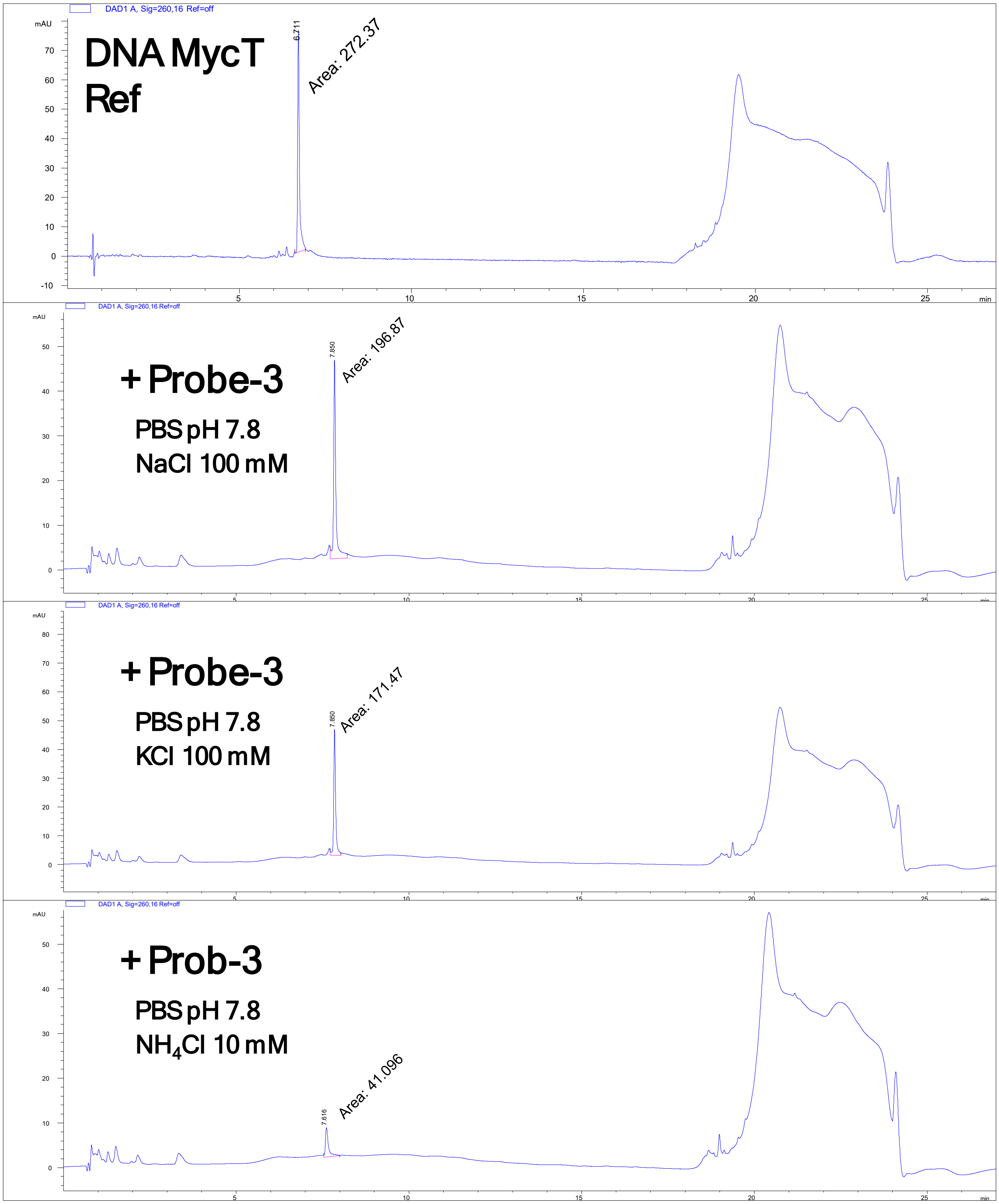

Figure S17. Pulldown experiment of DNA MycT in Probe-3. The experiments were performed at 5 µM strand concentration in PBS buffer 0.1M (10-100 mM salt concentration, pH 7.8).

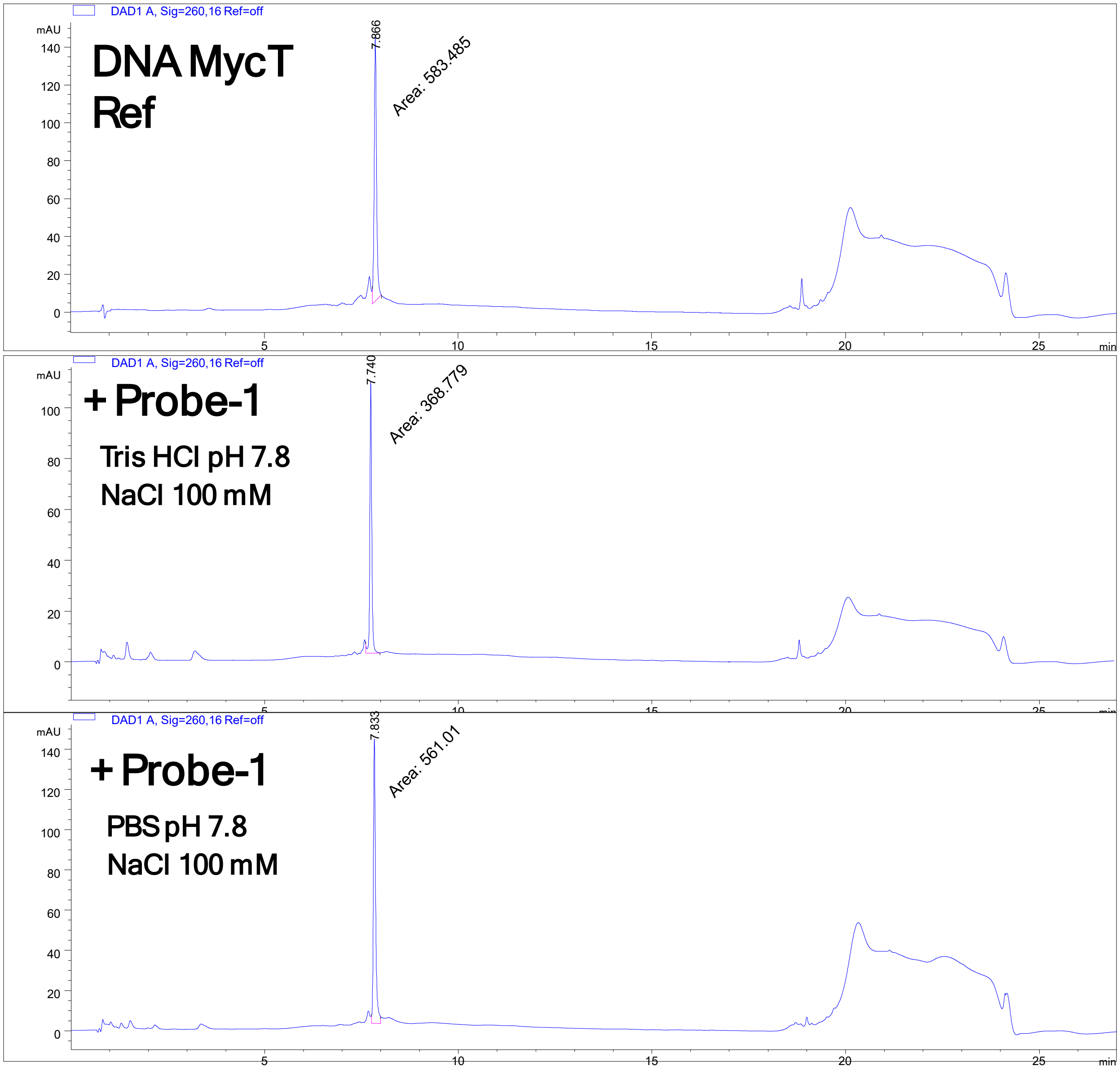

Figure S18. Pulldown experiment of DNA Myc T in presence of Probe-1. The experiments were performed at 5 µM strand concentration in Tris or PBS buffer 0.1M (100 mM NaCl, pH 7.8).

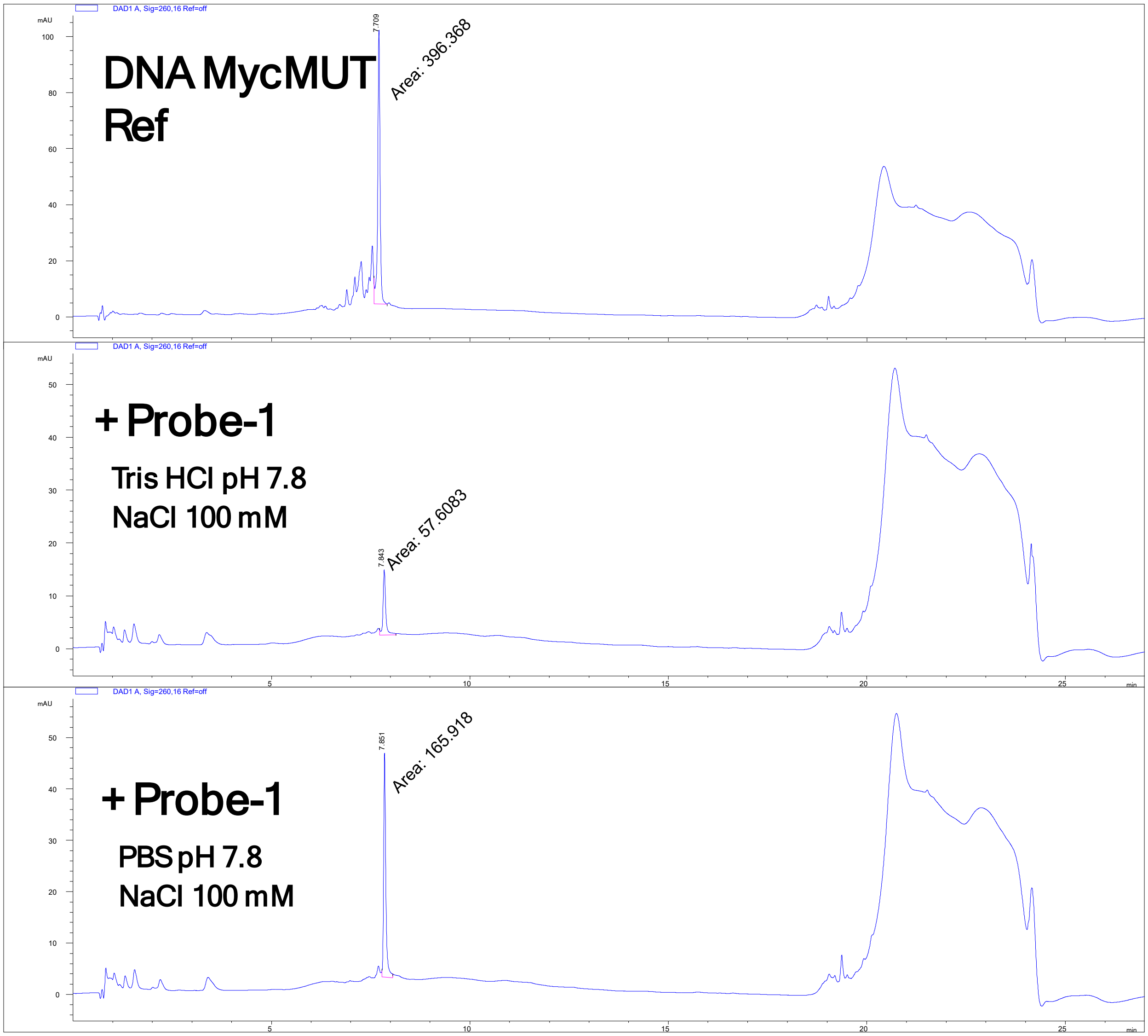

Figure S19. Pulldown experiment of DNA Myc Mut in presence of Probe-1. The experiments were performed at 5 µM strand concentration in Tris or PBS buffer 0.1M (100 mM NaCl, pH 7.8).

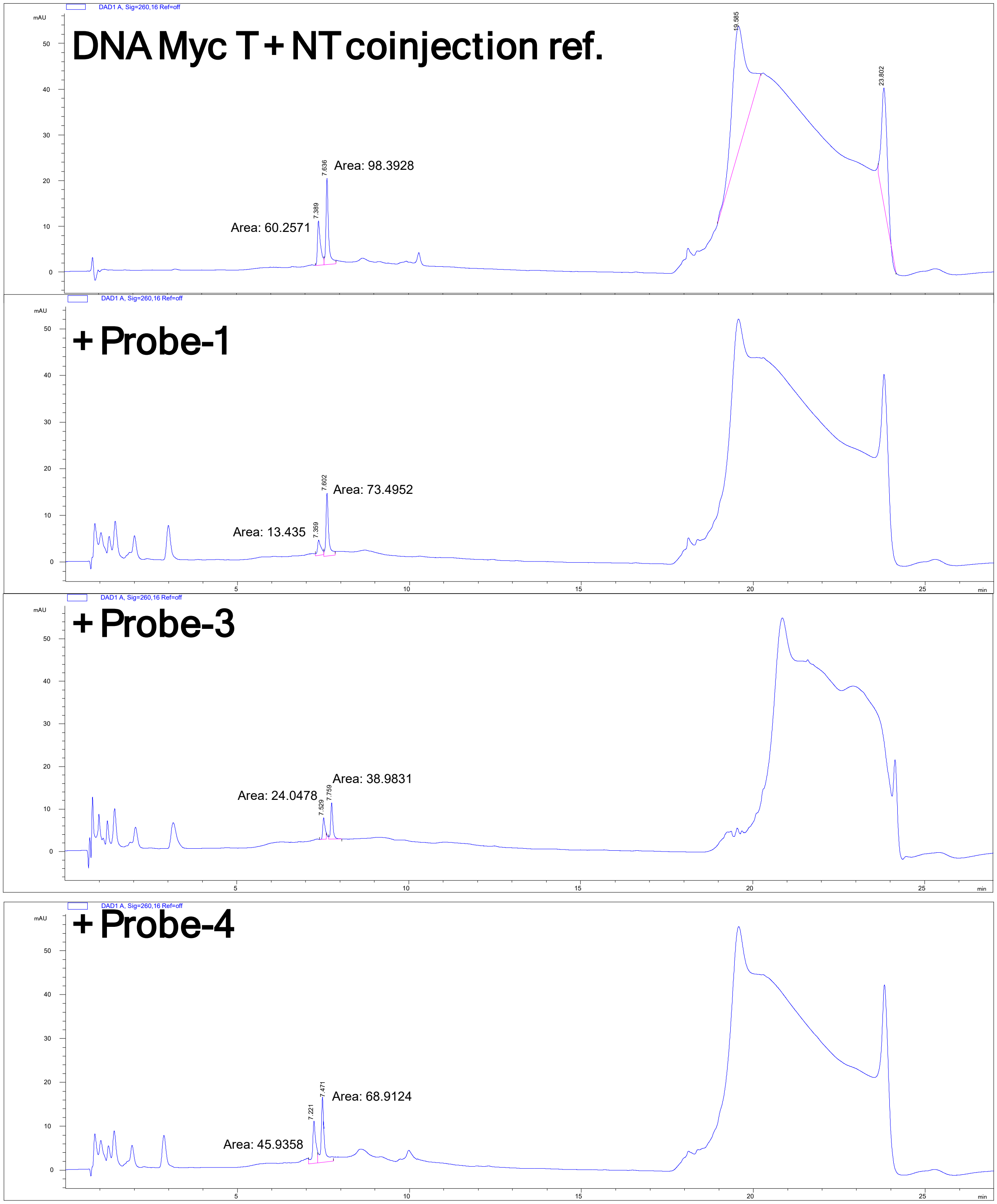

Figure S20. Pulldown experiment of DNA MycT + Myc NT in presence of Probe-1, Probe-3 and Probe-4. The experiments were performed at 5 µM strand concentration in PBS buffer 0.1M (100 mM NaCl, pH 7.8).

Pull-down and quantification using 96well plate

For the pull-down experiments analysed through UV spectroscopy (TMB oxidation), each solution coming from the pull-down (please refer to the material and methods section in the main paper) was transferred to a 96 well plate (NUNC) and analysed using a Trinean DropQuant UV plate reader after the TMB oxidation (please refer to the main paper for the full procedure). The concentration was calculated based on the calibration curve determined before each experiment.

Limit of Detection (LOD) was calculated using the formula:

$$LOD = \frac{3.3 \cdot \sigma}{m} \quad (1)$$

Where σ is the standard deviation of the response and m is the slope of the calibration curve. Both values were obtained using the built-in linear regression fitting function of GraphPad Prism.

A)

MYC T conc (µM)	*Abs 1*	*Abs2*
1	6.31	5.69
0.5	3.49	4.02
0.25	1.44	1.82
0.13	0.53	0.66
0.06	0.21	0.4
0.03	0.2	0.10
0	0.0	0.0

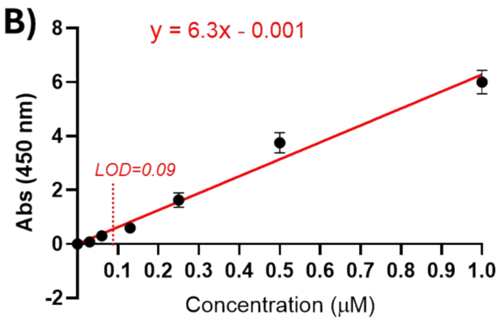

Figure S21. Example of calibration curve building from the raw absorbance data (A) obtained from linear dilution and (b) example of a calibration curve and LOD calculations.

Table S2. Raw absorption values obtained after the pulldown of DNA sequences with **Probe-1.**

DNA	Absorbance 1	Absorbance 2	Calculated Conc (µM)
MYC T	3.212	3.59	0.54 ± 0.04
MYC T, Dil	0.692	0.881	0.125 ± 0.02
c-KIT	0.2762	0.3707	0.0515 ± 0.01
c-KIT, Dil	-0.02494	0.00593	-0.00135 ± 0.003
kRAS	1.196	1.385	0.205 ± 0.02
kRAS, Dil	0.2699	0.2573	0.0420 ± 0.001

Table S3. Raw absorption values obtained after the pulldown of DNA sequences with **Probe-5.**

DNA	Absorbance 1	Absorbance 2	Calculated Conc (µM)
MYC T	2.834	3.086	0.47 ± 0.03
MYC T, Dil	0.503	0.566	0.085 ± 0.01
c-KIT	1.07	1.322	0.19 ± 0.03

c-KIT, Dil	0.0683	0.1061	0.014 ± 0.004
kRAS	2.645	3.023	0.45 ± 0.04
kRAS, Dil	0.692	0.691	0.11 ± 0.001

Supporting References

1 E. Cadoni, L. De Paepe, G. Colpaert, R. Tack, D. Waegeman, A. Manicardi and A. Madder, *Nucleic Acids Res.*, 2023, **51**, 4112–4125.

2 J. J. Díaz-Mochón, L. Bialy and M. Bradley, *Org. Lett.*, 2004, **6**, 1127–1129.
